# Pathway‐based integration of multi‐omics data reveals lipidomics alterations validated in an Alzheimer's disease mouse model and risk loci carriers

**DOI:** 10.1111/jnc.15719

**Published:** 2022-12-12

**Authors:** Monica Emili Garcia‐Segura, Brenan R. Durainayagam, Sonia Liggi, Gonçalo Graça, Beatriz Jimenez, Abbas Dehghan, Ioanna Tzoulaki, Ibrahim Karaman, Paul Elliott, Julian L. Griffin

**Affiliations:** ^1^ Department of Brain Sciences Imperial College London London UK; ^2^ Section of Biomolecular Medicine, Department of Metabolism, Digestion and Reproduction Imperial College London London UK; ^3^ UK‐Dementia Research Institute (UK‐DRI) at Imperial College London London UK; ^4^ Section of Bioinformatics, Department of Metabolism, Digestion and Reproduction Imperial College London London UK; ^5^ Section of Bioanalytical Chemistry and the National Phenome Centre, Department of Metabolism, Digestion and Reproduction Imperial College London London UK; ^6^ Department of Epidemiology and Biostatistics Imperial College London London UK; ^7^ MRC Centre for Environment and Health Imperial College London London UK; ^8^ National Institute for Health Research Imperial Biomedical Research Centre Imperial College London UK; ^9^ Department of Hygiene and Epidemiology University of Ioannina Medical School Ioannina Greece; ^10^ Department of Biochemistry and Cambridge Systems Biology Centre University of Cambridge Cambridge UK; ^11^ The Rowett Institute, University of Aberdeen Aberdeen Scotland

**Keywords:** Alzheimer's disease, ATP‐binding‐cassette subfamily‐a member‐7 gene (ABCA7), lipidomics, metabolome‐wide association study (MWAS), multi‐omics, pathway‐based integration

## Abstract

Alzheimer's disease (AD) is a highly prevalent neurodegenerative disorder. Despite increasing evidence of the importance of metabolic dysregulation in AD, the underlying metabolic changes that may impact amyloid plaque formation are not understood, particularly for late‐onset AD. This study analyzed genome‐wide association studies (GWAS), transcriptomics, and proteomics data obtained from several data repositories to obtain differentially expressed (DE) multi‐omics elements in mouse models of AD. We characterized the metabolic modulation in these data sets using gene ontology, transcription factor, pathway, and cell‐type enrichment analyses. A predicted lipid signature was extracted from genome‐scale metabolic networks (GSMN) and subsequently validated in a lipidomic data set derived from cortical tissue of ABCA‐7 null mice, a mouse model of one of the genes associated with late‐onset AD. Moreover, a metabolome‐wide association study (MWAS) was performed to further characterize the association between dysregulated lipid metabolism in human blood serum and genes associated with AD risk. We found 203 DE transcripts, 164 DE proteins, and 58 DE GWAS‐derived mouse orthologs associated with significantly enriched metabolic biological processes. Lipid and bioenergetic metabolic pathways were significantly over‐represented across the AD multi‐omics data sets. Microglia and astrocytes were significantly enriched in the lipid‐predominant AD‐metabolic transcriptome. We also extracted a predicted lipid signature that was validated and robustly modeled class separation in the ABCA7 mice cortical lipidome, with 11 of these lipid species exhibiting statistically significant modulations. MWAS revealed 298 AD single nucleotide polymorphisms‐metabolite associations, of which 70% corresponded to lipid classes. These results support the importance of lipid metabolism dysregulation in AD and highlight the suitability of mapping AD multi‐omics data into GSMNs to identify metabolic alterations.

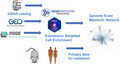

## Graphical abstract

A genome‐scale model network was built for Alzheimer's disease (AD) using data from publicly available genome‐wide association studies in humans, and transcriptomics and proteomics data from brain tissue from mouse models of AD. The derived network was characterized by gene ontology, transcription factor analysis, and pathway and cell‐type enrichment analysis. Lipid and bioenergetic pathways were over‐represented in the network, with microglia and astrocytes showing over‐enrichment of pathways. These network changes were validated in a mouse model of late‐onset AD (the ABCA7 null mouse) and a human metabolome‐wide association study (MWAS) of SNPs associated with late‐onset AD.

**Abbreviations**
ABCA7: ATP‐binding‐cassette subfamily‐A member‐7 gene
AD: Alzheimer's disease
Airwave: Airwave Health Monitoring Study
APOE: apolipoprotein epsilon
APP: amyloid precursor protein
Aβ: amyloid‐beta
ChEA3: ChIP‐X enrichment analysis 3
DAVID: database for annotation, visualization, and integrated discovery
DE: differentially expressed
EWCE: expression‐weighted cell‐type enrichment
FC: fold change
FDR: false discovery rate
GEO: gene expression omnibus
GRCh37: genome reference consortium‐human build‐37
GSMN: genome‐scale metabolic networks
GWAS: genome‐wide association studies
IGAP: international genomics of Alzheimer's cohorts
iTRAQ: isobaric tag for relative and absolute quantification
KO: knockout
MAGMA: multi‐marker analysis of genomic annotation
MWAS: metabolome‐wide association study
NMR: nuclear magnetic resonance
OPLS‐DA: orthogonal projections to latent structures‐discriminant analysis
PQN: probabilistic quotient normalization
PRIDE: protein identification database
RP‐UPLC‐MS: reverse‐phase ultraperformance liquid chromatography‐mass spectrometry
RS: Rotterdam study
S.D.f.M: standard deviation from the bootstrapped mean
SAM: significance analysis of microarray
SNPs: single nucleotide polymorphisms
SREBP2: sterol regulatory element‐binding protein 2
TF: transcription factor
TREM2: triggering receptor expressed on myeloid cells‐2
UPLC‐MS: ultraperformance liquid chromatography‐mass spectrometry
VIP: variable influence of projection
WT: wild type

## 1 INTRODUCTION

Alzheimer's disease (AD) is a neurodegenerative disorder prevalent in later life characterized by amyloid deposition, hyperphosphorylated tau aggregation into neurofibrillary tangles, and sustained neuroinflammatory response (Canchi et al., 2019). With the proportion of the population over 65 years of age increasing annually, a mechanistic understanding of the disease is urgently needed (Xie et al., 2020). There are several emerging lines of evidence highlighting the importance of metabolic dysfunctions in AD. Impaired glycolysis and bioenergetics shifts toward fatty acid and amino acid metabolism seem to indicate that mitochondrial dysfunction or substrate switch plays a role in AD pathogenesis (Wang et al., 2020). Cholesterol metabolism has also been shown to exert lipotoxic effects in the AD brain (Chan et al., 2012). Furthermore, there are several genes linked to AD onset and progression that are also related to brain lipid metabolism. The apolipoprotein epsilon4 (APOE4) allele, the strongest risk factor for AD development, is known to cause significant disruptions in brain lipid homeostasis in both human carriers and transgenic animals (Feringa & van der Kant, 2021). Similarly, triggering receptor expressed on myeloid cells‐2 (TREM2), another gene strongly associated with AD, actively undergoes lipid‐sensing and consequently induces changes in the microglia lipidome (Nugent et al., 2020). Finally, the loss‐of‐function variant of the ATP‐binding‐casette, subfamily‐A, member‐7 gene (ABCA7) was found to be strongly associated with late‐onset AD (De Roeck et al., 2019). ABCA7 is implicated in AD pathology through amyloid precursor protein (APP) endocytosis, impaired amyloid‐beta (Aβ) clearance and, although not fully elucidated, lipid metabolism dysregulation via sterol regulatory element‐binding protein 2 (SREBP2) (Aikawa et al., 2018).

Despite all the accumulating evidence, mechanistic explanations of AD have mostly been centered around amyloid or tau‐centric hypotheses, and therefore much remains to be understood regarding the underlying metabolic processes (Johnson et al., 2020).

Multi‐omics approaches have the potential to overcome the limitations of the current knowledge in this field. These approaches can provide a comprehensive view of a particular pathophysiological state by interrogating molecular changes across several levels of biological functions (Canzler et al., 2020). A promising methodological approach relevant to the study of metabolites is genome‐scale metabolic network (GSMN), which uses genomics and transcriptomics data to predict metabolic pathway modulations (Pinu et al., 2019). GSMN also allows for the interpretation of multi‐omics data via metabolic subnetwork curation, thus providing an attractive metabolic framework that can be effectively validated using metabolomics and lipidomics data (Frainay & Jourdan, 2017).

**FIGURE 1:**
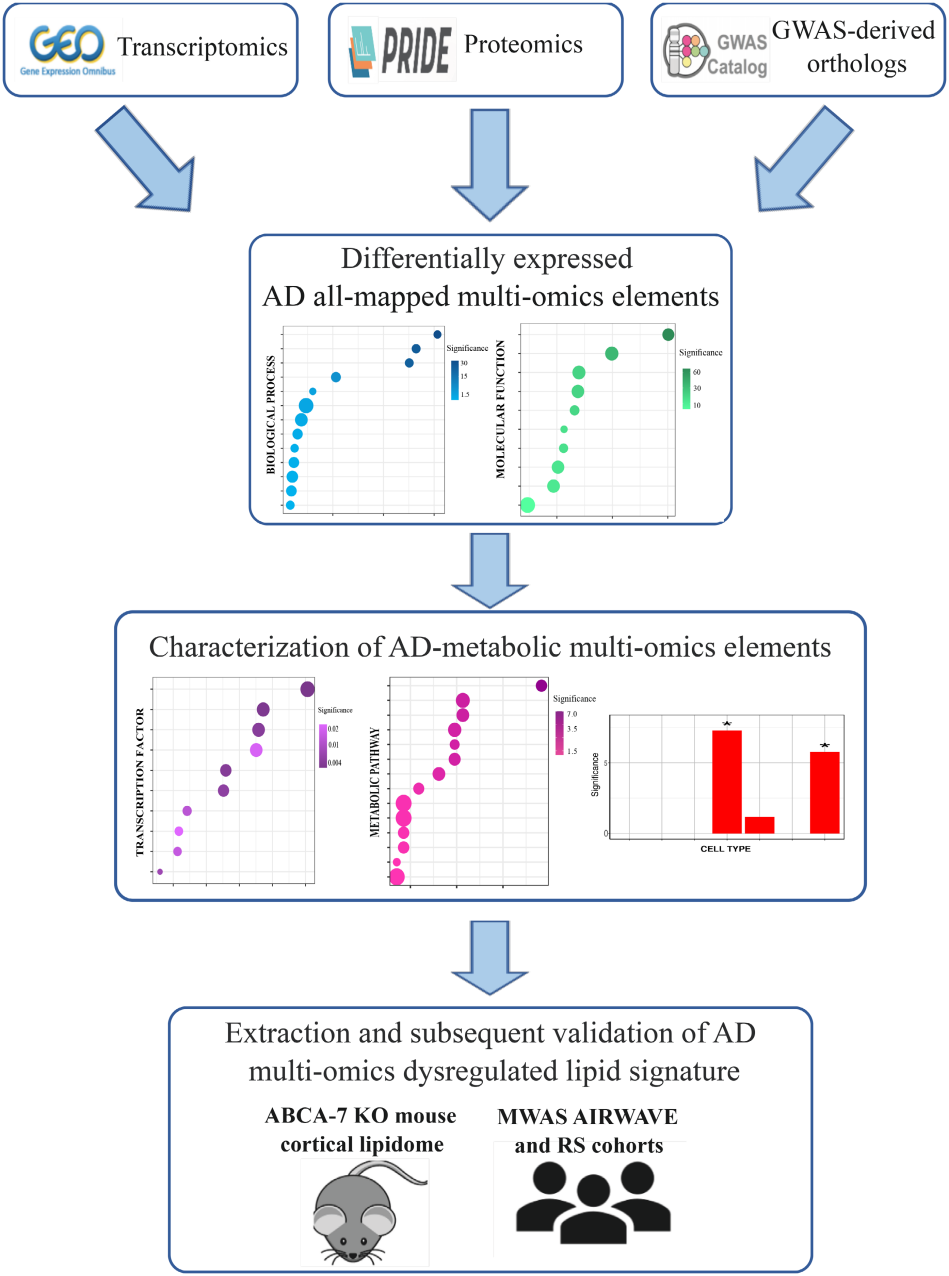
Schematic representation of the experimental design implemented in this study. ABCA7 KO, ATP‐binding‐cassette, subfamily a, member 7 gene knockout; AD, Alzheimer's disease; GEO, gene expression omnibus database; PRIDE, protein identification database

The aim of this study was to validate the presence of metabolic perturbations in AD using multi‐omics pathway‐based integration and extraction of metabolic subnetworks from open source data (Figure 1). We found consistent perturbations of lipid and energy metabolism across three AD multi‐omics data sets compiled from previous studies, from which we extracted 133 lipid species predicted to be dysregulated in AD which we then validated in an ABCA7 knockout (KO) mouse data set acquired with ultraperformance liquid chromatography‐mass spectrometry (UPLC‐MS). The importance of this association was explored further by performing a metabolome‐wide association study (MWAS) of the blood plasma metabolome for AD risk loci carriers in two human cohorts using ^1^H NMR spectroscopy.

This study also highlights the suitability of interpreting multi‐omics data in the context of GSMNs, as the predicted lipid terms and species were not only found in the cortical ABCA7 lipidome, but its associated multivariate model robustly separated ABCA7 mice from their wild‐type (WT) littermates.

## 2 MATERIALS AND METHODS

### 2.1 Data collection of AD mouse brain transcriptomics and proteomics data

The gene expression omnibus (GEO) repository (https://www.ncbi.nlm.nih.gov/geo/) (Clough & Barrett, 2016) was queried on June 15, 2020 for gene expression studies using “Alzheimer's disease” as a search term. The following criteria were employed for data set selection: *Mus musculus* organism, expression profiling by the array as study type, tissue as attribute, brain tissue expression compared with WTs, and a minimum of three animals per condition. This search yielded 11 data sets (GSE25926, GSE53480, GSE60460, GSE77574, GSE77373, GSE109055, GSE111737, GSE113141, GSE141509, and GSE74441) from nine studies (Aydin et al., 2011; Faivre et al., 2018; Fang et al., 2019; Hamilton et al., 2015; Hou et al., 2018; Marsh et al., 2016; Polito et al., 2014; Preuss et al., 2020; Wang et al., 2017).

The proteomics identification (PRIDE) repository (Jones et al., 2006) was queried on July 1, 2020 for proteomics studies applying the following filters: Alzheimer's disease as a disease, brain as organism part, and *Mus musculus* as organism. Data sets comparing the AD proteome against WTs, with a minimum of three animals per condition and with deposited proteinGroups.txt files were included. This search yielded four data sets (PXD007795, PXD011068, PXD012238, and PXD007813) from four publications (Hamezah et al., 2019; Kim et al., 2019; Lachen‐Montes et al., 2019; Palomino‐Alonso et al., 2017). However, differences in protein expression failed to reach statistical significance after controlling for the false discovery rate (FDR) in two studies (Hamezah et al., 2019; Palomino‐Alonso et al., 2017), and thus their corresponding data sets were excluded. A description of all included data sets can be found in Table 1.

**TABLE 1:** Characteristics of the transcriptomics and proteomics data sets included in this study

Brain region	GEO/PRIDE accession number	AD animal model	AD model mutations	Age	Sample size	Platform
Transcriptomics						
Frontal cortex	GSE113141	APP/PS1	APP_SWE_/ PS1_DE9_	9–10 months	AD (*n* = 6) WT (*n* = 6)	Agilent‐074809 SurePrint G3 Mouse GE v2 8x60K Microarray
	GSE109055	3xTgAD	PS1_M146V_/ APP_SWE_/ Tau_P301L_	22–24 months	AD (*n* = 4) WT(*n* = 4)	Agilent‐028005 SurePrint G3 Mouse GE 8x60K Microarray
	GSE77373	5xFAD	APP_SWE,_ _FLO, LON_/ PS1_M146L,_ L286V	5 months	AD (*n* = 3) WT(*n* = 3)	Affymetrix Mouse Gene 1.0 ST Array
	GSE74441	APP/PS1	APP_SWE_/ PS1_DE9_	Not disclosed	AD (*n* = 6) WT (*n* = 6)	Illumina MouseRef‐8 v2.0 expression beadchip
	GSE25926	APP‐KI	APP‐sα knock‐in	24–28 months	AD (*n* = 3) WT(*n* = 3)	Affymetrix Mouse Genome 430 2.0 Array
Hippocampus	GSE111737	APP/PS1	APP_SWE_/ PS1_DE9_	8 months	AD (*n* = 6) WT (*n* = 6)	Agilent‐074809 SurePrint G3 Mouse GE v2 8x60K Microarray
	GSE109055	3xTgAD	PS1_M146V_/ APP_SWE_/ Tau_P301L_	22–24 months	AD (*n* = 4) WT(*n* = 4)	Agilent‐028005 SurePrint G3 Mouse GE 8x60K Microarray
	GSE53480	rTg4510	Tau_P301L_	4 months	AD (*n* = 4) WT(*n* = 4)	Affymetrix Mouse Genome 430 2.0 Array
Subventricular zone	GSE60460	3xTgAD	PS1_M146V_/ APP_SWE_/ Tau_P301L_	7 months	AD (*n* = 4) WT(*n* = 4)	Agilent‐028005 SurePrint G3 Mouse GE 8x60K Microarray
Half‐brain	GSE141509	5xFAD	APP_SWE,_ _FLO, LON_/ PS1_M146L,_ L286V	6 months	AD (*n* = 6) WT (*n* = 6)	NanoString nCounter® Mouse AD panel
Whole brain	GSE77574	5xFAD	APP_SWE,_ _FLO, LON_/ PS1_M146L,_ L286V	6–7 months	AD (*n* = 4) WT(*n* = 4)	Affymetrix Mouse Transcriptome Array 1.0
Proteomics						
Hippocampus	PXD012238	5xFAD	APP_SWE,_ _FLO, LON_/ PS1_M146L,_ L286V	10 months	AD (*n* = 6) WT (*n* = 6)	Orbitrap MS/MS‐ Q‐Exactive
Olfactory bulb	PXD007813	Tg2576	APP_SWE_	6 months	AD (*n* = 3) WT(*n* = 3)	iTRAQ‐LC MS/MS with Triple TOF MS 5600

*Note*: GEO, gene expression omnibus repository (https://www.ncbi.nlm.nih.gov/geo/); PRIDE, proteomics identifications repository (Jones et al., 2006); AD, Alzheimer's disease; WT wild type; 3×TgAD, triple transgenic AD mice; 5×FAD, transgenic AD mice carrying 5 AD‐linked mutations; APP_*SWE*_, the KM670/671NL (Swedish) mutation in the amyloid precursor protein (APP) gene; PS1_*DE9*_, the deltaE9 mutation in the presenilin‐1 (PSEN1) gene; PS1_*M146V*_, the M146V mutation in the PSEN1 gene; Tau_*P301L*_, P301L mutation in the microtubule‐associated protein tau (MAPT) gene; APP_*SWE*_,_*FLO*,*LON*_, the Swedish, I716V (Florida) and V717I (London) mutations in the APP gene; PS1_*M146L,L286V*_, the M146L and L286V mutations in the PSEN1 gene; APP‐sα knock‐in, mutations in the stop codon behind the α‐secretase site of the APP gene.

### 2.2 Differentially expressed (DE) analysis of AD mouse transcriptomics and proteomics data

Processed transcriptomics data sets were retrieved from the GEO repository using the *GEOquery* Bioconductor‐based package (version 2.54.1) (Davis & Meltzer, 2007) in the R environment, version 3.6.2 (https://www.R‐project.org/). Data sets were log‐2 transformed and graphically inspected to verify appropriate data normalization; probes that were not mapped to any genes, mapped to more than one gene and probes with missing values (N/As) were filtered out. DE analysis was performed using significance analysis of microarray (SAM) with *samr* package (version 3.0) (Tusher et al., 2001) within the R environment. SAM can control for the total number of false positives through both gene‐specific t tests and a maximum local tolerable FDR (Tusher et al., 2001). Upon 200 permutation‐based SAM analysis, multiple testing correction was applied by adjusting the total false positives to 3% and the local FDR for the 90th percentile of DE genes to 5% in every data set.

Proteomics data sets were analyzed using Perseus (version 1.6.5) (Tyanova et al., 2016). Initially, proteins only identified by reverse‐decoy, site, or known contaminants were excluded, as well as proteins with 2/3 of replicates per group reporting N/As. Protein intensities were then log‐2 transformed and remaining N/As were replaced using normal distribution values, as most proteomics studies assume N/As are indicative of low‐expression proteins (Tyanova et al., 2016). DE proteins were determined using a two‐tailed Student's t test with a 200 FDR permutation‐based method and a 0.050 *p*‐value cutoff (Tusher et al., 2001). In isobaric tag for relative and absolute quantification (iTRAQ) experiments, an additional fold change (FC) 1.17–0.83 cutoff was introduced to determine DE proteins. iTRAQ experiments are prone to interference/ratio distortion (Pappireddi et al., 2019), and thus a combination of *p*‐value, FDR, and FC cutoff is the most suitable approach to detect biological variability (Oberg & Mahoney, 2012).

### 2.3 AD genome‐wide association studies (GWAS) gene‐based analysis and mouse ortholog determination

AD GWAS summary statistics were obtained from a meta‐analysis of the UK‐Biobank and International Genomics of Alzheimer's Project (IGAP) cohorts, which evaluated GWAS with AD by proxy in 388 364 individuals across both cohorts (Marioni et al., 2018). Summary statistics (ID: GCST005922) were retrieved from the NHGRI‐EBI GWAS Catalog (https://www.ebi.ac.uk/gwas/) (Buniello et al., 2019) on 07/07/2020.

Gene‐based analysis was performed with multi‐marker analysis of genomic annotation (MAGMA) (version 1.07bb) (de Leeuw et al., 2015), using gene locations from the genome reference consortium‐human build‐37 (GRCh37, NCBI) and a reference panel of European ancestry from the 1000 genomes project phase‐3 (1000 Genomes Project Consortium, Auton et al., 2015). MAGMA provides a combined p‐statistic of genes significantly associated with single nucleotide polymorphisms (SNPs) (de Leeuw et al., 2015); we used a combined 0.050 *p*‐value as a significance cutoff. Significant genes were imported into Ensembl–Biomart on July 20, 2020 (version GrCh37.13, https://grch37.ensembl.org/biomart/martview) (Zerbino et al., 2018) to determine high‐confidence mouse orthologs. Upon excluding genes associated with either several or no mouse orthologs, only those exhibiting one‐to‐one bidirectional orthology with 60% protein sequence similarity across both species were considered high‐quality mouse orthologs (Mancuso et al., 2019).

### 2.4 Gene ontology (GO) analysis and AD‐metabolic multi‐omics extraction

DE transcripts, proteins, and GWAS‐orthologs were initially mapped onto the BioCyc *Mus musculus* GSMN (Caspi et al., 2016) using MetExplore (Cottret et al., 2018), which provides a framework for metabolic subnetwork extraction. DE transcripts, protein‐coding, and GWAS ortholog genes that were not mapped onto the GSMN were removed; the resulting omics lists are referred to as “all‐mapped” data throughout this study. Significantly enriched functional terms were identified in all‐mapped AD omics data sets using the database for annotation, visualization, and integrated discovery (DAVID) (version v.6.8) (https://david.ncifcrf.gov/) (Dennis Jr. et al., 2003) and the *Mus musculus* genome as background. GO analysis was performed using a hypergeometric test with an EASE score of 0.1 and a count threshold of 2. Terms with both raw *p*‐value and Benjamini‐Hochberg (B‐H) FDR‐adjusted *p*‐value below 0.050 were considered statistically significant. Metabolism‐related transcripts, proteins, and GWAS‐orthologs were manually extracted from significantly enriched biological processes (BP).

### 2.5 Transcription factor (TF) enrichment analysis

TF enrichment analysis was performed on all‐mapped AD genes and proteins, as well as their metabolic counterparts, using ChIP‐X enrichment analysis 3 (ChEA3) (https://maayanlab.cloud/chea3/). ChEA3 performs enrichment analysis based on TF's target genes coverage using the Fishers exact test and B‐H adjusted *p*‐value at 0.050 thresholds (Keenan et al., 2019). The ENCODE library was chosen as our reference set, as it incorporates TF‐target associations from human and mouse data (Davis et al., 2018). Significantly enriched TF were manually cross‐referenced with the mouse transcription factor atlas to verify its mouse tissue expression (Zhou et al., 2017).

### 2.6 Pathway enrichment analysis of AD‐metabolic multi‐omics data

AD‐metabolic transcripts, proteins, and GWAS‐ortholog lists were mapped onto the BioCyc *Mus musculus* GSMN (Caspi et al., 2016) in MetExplore (Cottret et al., 2018). Metabolic pathway enrichment analysis was performed using hypergeometric tests with right‐tailed Fisher's exact tests with B‐H correction for multiple testing (α = 0.050).

### 2.7 Expression‐weighted cell‐type enrichment (EWCE) of AD‐metabolic multi‐omics data

EWCE was conducted on AD‐metabolic transcriptomics, proteomics, and GWAS‐ortholog data sets using the *EWCE* package in R (version 0.99.2) (Skene & Grant, 2016). EWCE computes an enrichment *p*‐value that describes the probability of an input gene list having a meaningful expression within a specific cell type upon 10 000 random permutations (Skene & Grant, 2016). A cortical and hippocampal single‐cell RNA‐sequencing data set with large coverage was used as background (Zeisel et al., 2015); B‐H adjusted *p*‐values were calculated using the R base package. A conditional EWCE analysis was also performed on the combined AD‐metabolic multi‐omics data set to probe the relationships between enriched cell types, using an approach originally developed for GWAS data analysis (Skene et al., 2018).

### 2.8 Metabolic subnetwork extraction

To ultimately validate lipid alterations highlighted during pathway enrichment analysis, a metabolic subnetwork containing all lipid terms or species in significantly enriched lipid pathways were mined across the AD‐metabolic transcriptome and proteome using MetExplore (Cottret et al., 2018). After excluding non‐lipid metabolites, a combined predicted lipid signature across the AD multi‐omics data sets was created, which was visualized using MetExploreViz (Chazalviel et al., 2018). Lipid identifiers were then retrieved from LIPID MAPS (Fahy et al., 2009).

### 2.9 Cortical ABCA7‐KO lipidomics data set

We also acquired a novel lipidomics data set of cortical extracts of 7 WT and 7 ABCA7‐KO 11‐month‐old mice, with 3 females and 4 males per group. The genetic background of this AD animal model has been described previously (Aikawa et al., 2018). Lipidomic extraction was performed on ~50 mg cortex tissue using modified Folch extraction (Su et al., 2019). Global lipidomic profiling of the cortical extracts and 3 pooled samples was acquired using a reverse‐phase ultraperformance liquid chromatography‐mass spectrometry (RP‐UPLC‐MS) on a Synapt Quadruple‐Time of Flight mass spectrometer (Waters Corp.) in positive and negative modes. Details of system configurations and analytical conditions have been previously reported (Andreas et al., 2020).

Data processing was performed using KniMet (Liggi et al., 2018). Briefly, signals extracted using the R library XCMS (Tautenhahn et al., 2012) were retained if present in at least 50% of the pooled samples with a coefficient of variation of ≤20. The remaining signals were subjected to the imputation of N/As using K‐Nearest Neighbor (KNN), probabilistic quotient normalization (PQN) based on pooled samples, and annotation using LIPID MAPS (https://lipidmaps.org) (Fahy et al., 2009), retention time matching to standards and fragmentation data.

### 2.10 Multivariate statistical analysis

Multivariate statistical analysis was performed on both positive and negative modes for the original ABCA7‐KO and validated lipid signature subsets using P‐SIMCA 14 (Umetrics, Sweden) following log‐transformation of intensities and Pareto‐scaling. Orthogonal projections to latent structures‐discriminant analysis (OPLS‐DA) models, which allow to evaluate the impact of group membership by separating the variance attributed or orthogonal to class membership into components, were created for both original data sets and validated subset in positive and negative ion modes (Griffin et al., 2020). Lipids in the validated subset in positive and negative modes with a variable influence of projection (VIP) > 1 were retained for univariate analysis, as OPLS‐DA generated VIP >1 indicates specific variables with important contributions to the model (Liu et al., 2020). The suitability of the models was assessed through inspection of their *R* ^2^ (cum)X and Q^2^ values, which, respectively, represent the percentage of model‐captured variation and predictive capability (Liu et al., 2020). Models were further validated with a 100 permutation‐based test in which the correlation coefficient for the permuted class‐membership variable is plotted against the R^2^ (cum)X and *Q* ^2^ (cum) (Murgia et al., 2017).

### 2.11 Univariate statistical analysis

AD multi‐omics lipid species that had an associated VIP score above 1 in the original ABCA7 KO lipidomics data set underwent univariate statistical analysis using GraphPad Prism and core functions in the R environment. Negative‐ and positive‐mode acquired lipids underwent both a Student *t* test and a Mann–Whitney non‐parametric test comparing genotype (*p* < 0.050); features were considered statistically significant if *p* < 0.050 across parametric testing and exhibiting relative fold changes above 0.5 compared with WT. Positive‐mode acquired lipids were also analyzed using one‐way ANOVA comparing genotype and sex correcting for multiple testing using the B‐H method (α < 0.05).

Hierarchical clustering coupled with Spearman's correlation among the significant metabolic features from negative‐mode open profiling lipidomics, as well as the data set subsets representing the validated AD multi‐omics lipid signature, were calculated and visualized using *corrplot* in R.

### 2.12 Metabolome‐wide association study (MWAS) of the blood plasma metabolome for AD risk loci carriers

We performed a novel MWAS to investigate the relationship between the human blood metabolome and AD risk. To do so, we used nuclear magnetic resonance (NMR) spectra of blood from 3258 individuals from the Airwave Health Monitoring Study (Airwave) and the Rotterdam Study (RS) prospective cohorts (Elliott et al., 2014; Ikram et al., 2020). These cohorts have been successfully employed to characterize the overall dementia burden in the elderly (Ikram et al., 2020) as well as investigate the associations between the human blood metabolome, cardiovascular disease, and biological aging (Robinson et al., 2020; Tzoulaki et al., 2019).

Blood samples were heparin plasma for Airwave and serum for RS. The average age at enrollment in 2004 was 40.9 years for men and 38.5 years for women in the Airwave cohort; the RS cohort mean age of recruitment was 55 for both genders in 1990 (Elliott et al., 2014; Ikram et al., 2020). Sample preparation and metabolic profiling in these cohorts have been extensively described (Robinson et al., 2020; Tzoulaki et al., 2019). Briefly, ^1^H NMR solvent suppression pulse and T2‐Carr‐Purcell‐Meiboom‐Gill (CPMG) spectra were acquired per sample (Dona et al., 2014) and additionally lipid quantification was applied on the ^1^H NMR solvent suppression pulse using a commercial package (Jiménez et al., 2018). Resonances associated with both protons attached to the fatty acid and the head group (largely choline and glycerol) along with protons from cholesterol and cholesterol esters were classified as belonging to the lipid class.

MWAS was then performed using 47 unique genetic loci based on three recent GWAS meta‐analysis on AD to identify AD risk loci carriers (Jansen et al., 2019; Kunkle et al., 2019; Lambert et al., 2013). These studies evaluated genome‐wide associations with late‐onset AD (LOAD) in individuals across the IGAP and UK‐Biobank cohorts.

### 2.13 MWAS association statistics

We carried out a linear regression to calculate the effect estimates of each SNP with all metabolomic features (23 571 data points for original NMR spectra and 105 features for the fitted lipid data) with adjustment for age, sex, and cohort. Prior to the analysis, each cohort data were residualised using 10 principal components from genome‐wide scans to adjust for population stratification. To account for multiple testing, we used a permutation‐based method to estimate the Metabolome‐Wide Significance Level (MWSL) to consider the high degree of correlation in metabolomics data sets (Castagné et al., 2017; Chadeau‐Hyam et al., 2010). A *p*‐value threshold giving a 5% Family‐Wise Error Rate was computed for each SNP in each data platform.

## 3 RESULTS

### 3.1 DE analysis of mapped AD mouse transcriptomics and proteomics data

DE transcripts and proteins in the AD mouse brain with potential metabolic functions were extracted from the GEO and PRIDE repositories, respectively. Microarray expression profiles from 11 data sets were obtained from 5 distinct brain regions (frontal cortex, hippocampus, subventricular zone, brain hemisphere, and whole brain) and 5 AD mouse models (APP/PS1, 5xFAD, 3xTgAD, APP‐KI, and Tg4510; Table 1). Of these, 7 (63.3% of all transcriptomics data sets) were obtained from AD mice exclusively harboring mutations in genes involved in amyloid processing (*APP* or *PS1*). The 4 remaining data sets were obtained from mice harboring additional (27.3% of all transcriptomics data sets) or only (9.4% of transcriptomics data sets) mutations in the *MAPT* gene, thus modeling amyloid deposition and tau aggregation. SAM revealed 2884 DE genes with a 90th percentile FDR below 5% (Table S1). Of these, 594 were accurately mapped onto the GSMN, which were used to generate the all‐mapped AD transcriptomics data set (Table S2). Furthermore, proteomics data sets from the hippocampus and olfactory bulb of 5xFAD and Tg2576 mice, harboring mutations in genes involved in amyloid and tau aggregation, were also obtained (Table 1). Permutation‐based analysis revealed 1537 DE proteins (FDR *p* < 0.050, Table S1), of which 392 were mapped onto the GSMN and therefore constituted the all‐mapped AD proteomics data set (Table S2). DE proteins from two additional studies (Hamezah et al., 2019; Palomino‐Alonso et al., 2017) failed to reach statistical significance upon FDR correction and thus these data sets were removed from further analysis.

### 3.2 Mapped high‐quality mouse orthologs identification from gene‐based AD GWAS analysis

High‐confidence mouse orthologs of significantly associated genes in human AD GWAS studies were also identified to gain a more comprehensive view of metabolic perturbations in AD. Gene‐based analysis with MAGMA (de Leeuw et al., 2015) using summary statistics from 388 364 individuals in the UK‐Biobank and IGAP cohorts (Marioni et al., 2018) revealed 18 178 gene‐level associations with human AD SNPs, of which 1664 were considered significant (combined *p*‐value <0.05). After applying high‐quality mouse orthology criteria (Mancuso et al., 2019), 1356 high‐quality orthologs of AD SNPs‐associated human genes were identified. The all‐mapped AD GWAS‐orthologs data set was generated by accurately mapping 258 GWAS‐orthologs onto the GSMN (Table S2).

### 3.3 Differential GO and TF enrichment analysis across AD multi‐omics data sets

**FIGURE 2:**
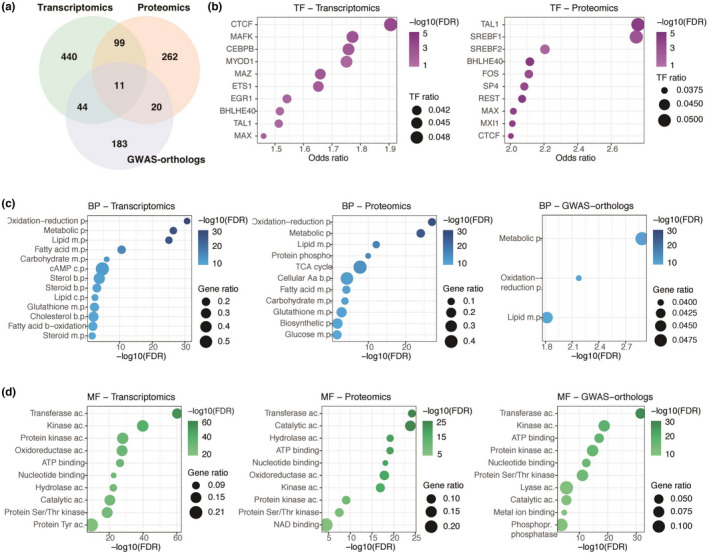
Transcription factor and functional enrichment analysis reveal shared functional processes between all‐mapped AD multi‐omics data sets (a) Venn diagram shows the amount of overlap between AD‐mapped transcripts, proteomics, and GWAS‐ortholog genes. (b) Top 10 TF enrichment analysis results of AD transcriptomics and proteomics data sets. (c) Selected biological process (BP) functional enrichment analysis of three AD multi‐omics data sets. “M.p,” “b.p.,” and “c.p.” refer to metabolic, biosynthetic, and catabolic processes, respectively. (d) Top 10 molecular functional (MF) enrichment analysis of three AD multi‐omics data sets. “Ac” refers to molecular function activity. TF ratio refers to the number of mapped input genes in relation to the total TF's target genes. –log10(FDR) refers to the inverse, log‐transformed FDR‐adjusted enrichment *p*‐value. Gene ratio refers to the number of mapped input genes in relation to all gene ontology (GO) term‐associated genes. The entire list of over‐represented TF and GO terms can be found in Tables S3 and S4, respectively.

Potential TF and GO enrichment were investigated across the AD multi‐omics data sets. More than 25% of differentially expressed, mapped AD protein‐coding genes were also found in the AD‐mapped transcriptomics data set, thus indicating a certain degree of consistency across our omics results (Figure 2a). Across our multi‐omics data sets, we found 67 TF to be significantly enriched in the all‐mapped AD proteome, whereas only 17 TF were significantly enriched in the all‐mapped AD transcriptome (Table S3). Despite these differences, *CCCTC‐binding factor* (*CTCF*), *TAL BHLH transcription factor 1* (*TAL1*), *MYC‐associated factor X* (*MAX*), and *basic helix‐loop‐helix family member E40* (*BHLHE40*) were among the top10 potential‐enriched TFs across both data sets (FDR *p* < 0.050, Figure 1b, Table S3).

GO analysis revealed shared functional terms across the three data sets (Figure 1c,d). Oxidation–reduction, lipid, and fatty acid metabolic processes were enriched in all‐mapped AD transcriptomics and proteomics (FDR *p* < 0.050, Figure 1c). Six additional lipid‐related BP terms were over‐represented in all‐mapped AD transcriptomics data, whereas the TCA cycle was only enriched in the AD proteome (FDR *p* < 0.050, Figure 1c). Transferase, catalytic, ATP‐binding, kinase activity, nucleotide binding, and serine/threonine‐kinase activity were among the top10 over‐represented terms across all‐mapped AD multi‐omics data sets (FDR *p* < 0.050, Figure 1d). Cytosol and mitochondria were the cellular compartment (CC) terms most over‐represented in the all‐mapped AD transcriptome and proteome, respectively; membrane was the only significant CC term in the AD GWAS‐orthologs data set (Table S4).

### 3.4 Lipid‐related metabolic pathways and regulators are enriched across AD‐metabolic multi‐omics data sets

**FIGURE 3:**
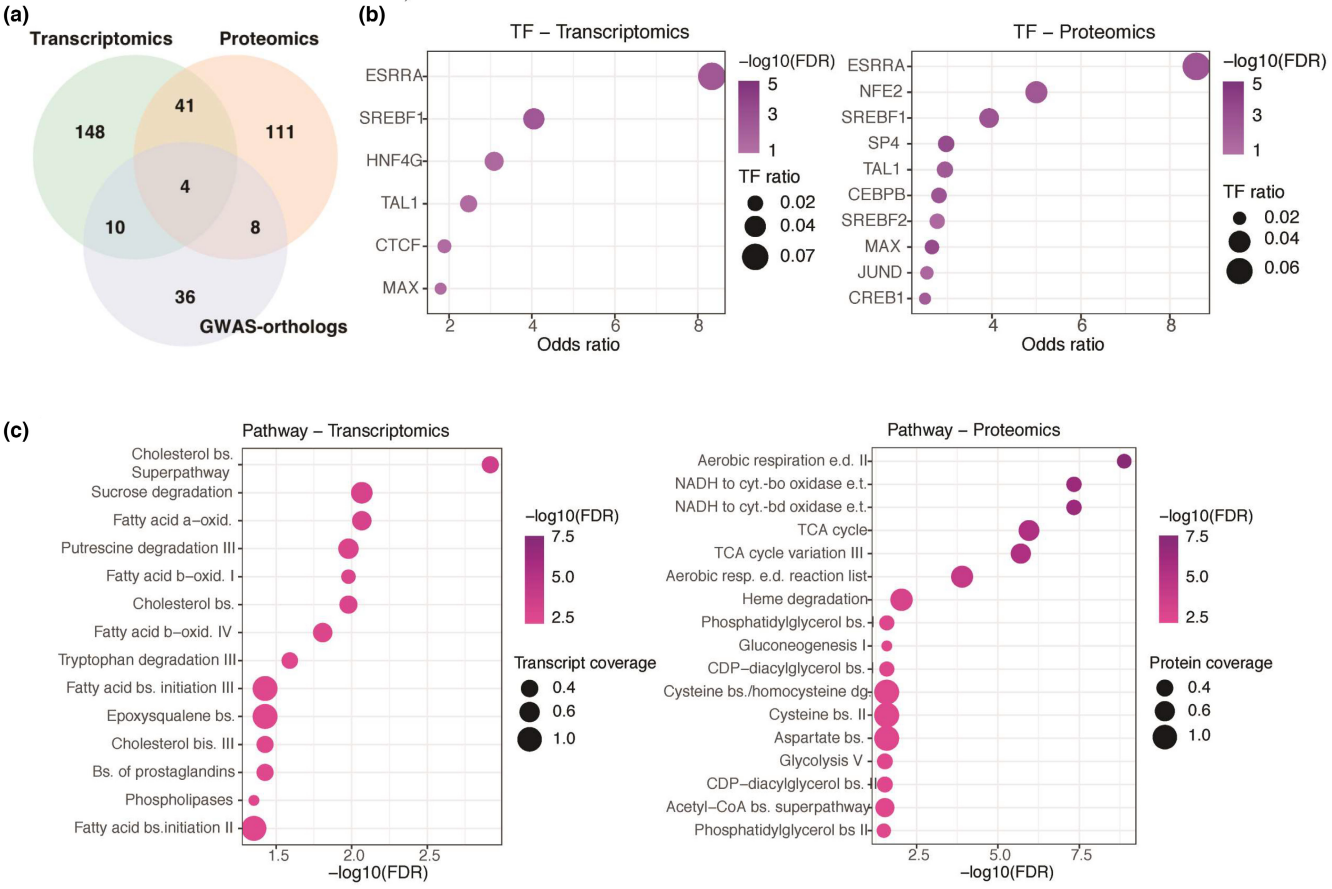
TF and pathway enrichment analysis highlight the enrichment of lipid‐related metabolic processes in AD‐metabolic transcriptomic and proteomic data sets. (a) Venn diagram shows the amount of overlap between AD‐metabolic multi‐omics data sets. (b) The top 10 TFs are significantly over‐represented in AD‐metabolic transcripts and proteins. (c) Pathway enrichment analysis of the three AD multi‐omics data sets. “Bs.,” “e.d.,” and “e.t.” refer to biosynthesis, electron donors, and electron transfer processes, respectively. –log10(FDR), the inverse, log‐transformed FDR‐adjusted enrichment *p*‐value; TF ratio, the number of mapped input genes in relation to the total TF's target genes; gene and protein cov., the number of mapped input elements in relation to all pathway‐mapped elements. The entire list of significantly enriched metabolic TF and pathways can be found in Tables S5 and S6.

Given the elevated number of metabolic BP significantly enriched across the three multi‐omics data sets, the DE 203 transcripts, 164 proteins, and 58 GWAS‐ortholog genes mapped to these BP were subjected to further characterization (Figure 3a, Table S2). The top 10 differentially expressed AD‐metabolic transcripts were *pde1c*, *idh2*, *aspg*, *mgst1*, *hk3*, *ndufa2*, *xdh*, *mecr*, and *npl* (Table S2). The top 10 differentially expressed AD‐metabolic gene‐encoding proteins were *gusb*, *agpat1*, *hexa*, *hexb*, *fdps*, *gfpt1*, *pld3*, *aldh1l1*, and *pdha1*; the top 10 DE AD‐metabolic GWAS‐orthologs were ndufs3, cyp51, aco2, hexb, hsd3b7, dbt, kdm2b, papss1, and ndufa2 (Table S2). The largest degree of overlap was again found between AD‐metabolic transcripts and proteins, with 41 genes differentially expressed in both data sets (Figure 3a). Although there were substantially more enriched TFs in the AD‐metabolic proteome (Table S5), lipid‐associated TFs such as *estrogen‐related receptor alpha (ESRRα)* and *sterol regulatory element‐binding transcription factor 1* (*SREBF1)* were over‐represented in the AD‐metabolic transcriptome and proteome (FDR *p* < 0.050, Figure 3b). Pathway enrichment analysis reflected differential metabolic processes across the multi‐omics data sets (Figure 3c). Pathways related to cholesterol, phospholipases, and fatty acid metabolism were significantly over‐represented in the AD‐metabolic transcriptomics data set, whereas the AD‐metabolic proteome was associated with mitochondrial processes such as TCA cycle, glycolysis, and NADH electron transfer (FDR *p* < 0.050, Figure 3c). Lipid processes such as CPD‐diacylglycerol and phosphatidylglycerol synthesis were also enriched in the AD‐metabolic proteome (Figure 3c). Thyroid hormone metabolism was significantly enriched in the GWAS‐orthologs data set with 66% pathway coverage (Table S6).

### 3.5 Astrocytes and microglia are independently enriched in the AD‐metabolic transcriptome

**FIGURE 4:**
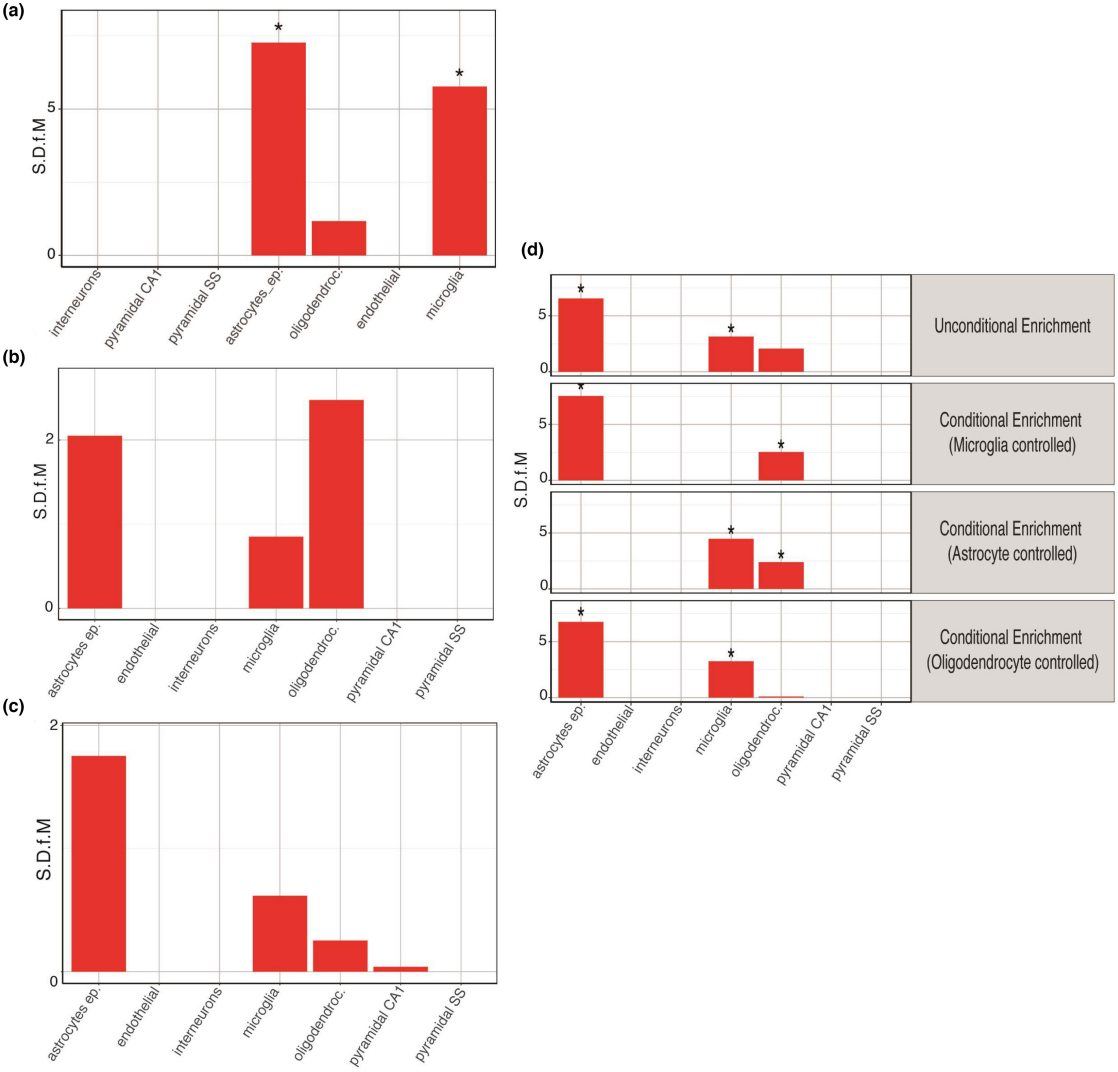
Cell‐type enrichment analysis of individual and combined AD‐metabolic multi‐omics data sets highlight independent astrocyte and microglia enrichment. Unconditional cell‐type enrichment analysis of AD‐metabolic (a) transcriptomics (b) proteomics and (c) GWAS‐orthologs data sets. (d) Conditional cell‐type enrichment analysis of combined AD multi‐omics data set. “S.D.f.M" indicates the standard deviation from the bootstrapped mean. The asterisk indicates statistical significance upon adjusting for FDR with the Benjamini‐Hochberg (B‐H) method (*p* < 0.050).

To determine whether cell‐type enrichment differences across the AD‐metabolic multi‐omics data sets could account for the differential pathway over‐representation described previously, unconditional EWCE was performed. Significant astrocyte (FDR *p*‐value = 0.0000001, standard deviation from the bootstrapped mean or S.D.f.M = 7.266), and microglia enrichment (FDR *p*‐value = 0.0000001, S.D.f.M = 5.770) was found in the AD‐metabolic transcriptomics data set (Figure 4a). Oligodendrocyte and astrocyte enrichment in the AD‐metabolic proteome lost significance upon multiple testing correction (FDR *p*‐value = 0.07, S.D.f.M = 2.474 and FDR *p*‐value = 0.095, S.D.f.M = 2.049, respectively, Figure 4b). Astrocyte enrichment was also similarly lost in the GWAS‐orthologs data set (FDR *p*‐value = 0.336, S.D.f.M = 1.75, Figure 4c).

Conditional cell‐type enrichment was performed on a combined AD‐metabolic multi‐omics data set to investigate enrichment relationships. Controlling for microglia did not ablate astrocytic enrichment (FDR *p*‐value = 0.0000001, S.D.f.M = 7.540) and vice‐versa (FDR *p*‐value = 0.0000001, S.D.f.M = 4.476), suggesting astrocyte and microglia enrichments were independent of each other (Figure 4d). Oligodendrocyte enrichment was, however, dependent on microglia and astrocytes, as significance was lost upon controlling for either of them (FDR *p*‐value = 0.0389, S.D.f.M = 2.531 and FDR *p*‐value = 0.0389, S.D.f.M = 2.387 respectively, Figure 4d). Cell‐type enrichment statistics can be found in Table S7.

### 3.6 Validation of AD multi‐omics lipid signatures in ABCA7‐KO mice cortex

The GSMN network analysis highlighted cholesterol, fatty acid, and glycerolipid metabolism as being altered in the AD‐metabolic transcriptome and proteome. These results were validated by comparing them to a newly acquired lipidomics UPLC‐MS data set from cortical extracts of ABCA7‐KO and WT mice.

To do so, we first extracted transcripts and protein‐coding genes mapped to significantly altered lipid metabolism pathways in the AD‐metabolic transcriptome and proteome. These pathways are annotated as WY66‐5, PWY‐2501, FAO‐PWY, PWY66‐341, PWY‐5138, PWY3DJ‐35 583, PWY3DJ‐35 583, PWY66‐4, PWY‐5670, PWY‐5965, PWY‐5966, LIPASYN‐PWY, PWY4FS‐7, PWY‐5667, PWY‐5173, PWY0‐1319, and PWY4FS‐8 in the BioCyc *Mus musculus* GSMN (Table S6). These transcripts and protein‐coding genes were used to create a metabolic subnetwork of the AD‐metabolic dysregulated lipid metabolism. This subnetwork involved 119 enzyme‐coding genes, 81 metabolic reactions, and 107 metabolites, of which 73 were either unique lipid species or lipid terms (lipid subclasses such as a CDP‐diacylglycerol). Hence, we extracted 49 unique lipid species using first‐order lipid identifier matching criteria. This included mevalonate (GSMN ID: MEVALONATE), 24,25‐dihydrolanosterol (GSMN ID: CPD‐8606), and 3‐keto‐4‐methylzymosterol (GSMN ID: CPD‐4578) in the data set (Table S8).

Furthermore, we also extracted 84 unique lipid species from 22 lipid terms using second‐order lipid identifier matching criteria. This included 1‐acyl‐sn‐glycero‐3‐phosphocholine, LPC 18:1, and LPC 20:3 all annotated to the GSMN lipid term ID: 1‐Acylglycero‐Phosphocholines (Table S8). The combination of first‐ and second‐order lipid term matching yielded 133 lipid species (Table S8), which we refer to as the generated predicted AD multi‐omics lipid signature.

Twenty‐eight terms and 60 lipid species from the predicted AD multi‐omics lipid signature were found and therefore validated in the ABCA7‐KO and WT lipidomes (Table S8). In particular, 40 lipid species were validated in the negative‐mode data set and 20 species in the positive‐mode data set. The original MS data, containing 5025 and 5811 features in positive and negative ionization modes, respectively, were hence filtered based on these two subsets of lipid species. OPLS‐DA was then performed on both original and filtered data sets to assess the presence of any possible separation based on gender and/or genotype, and the potential impact of this feature reduction procedure on the model robustness.

**FIGURE 5:**
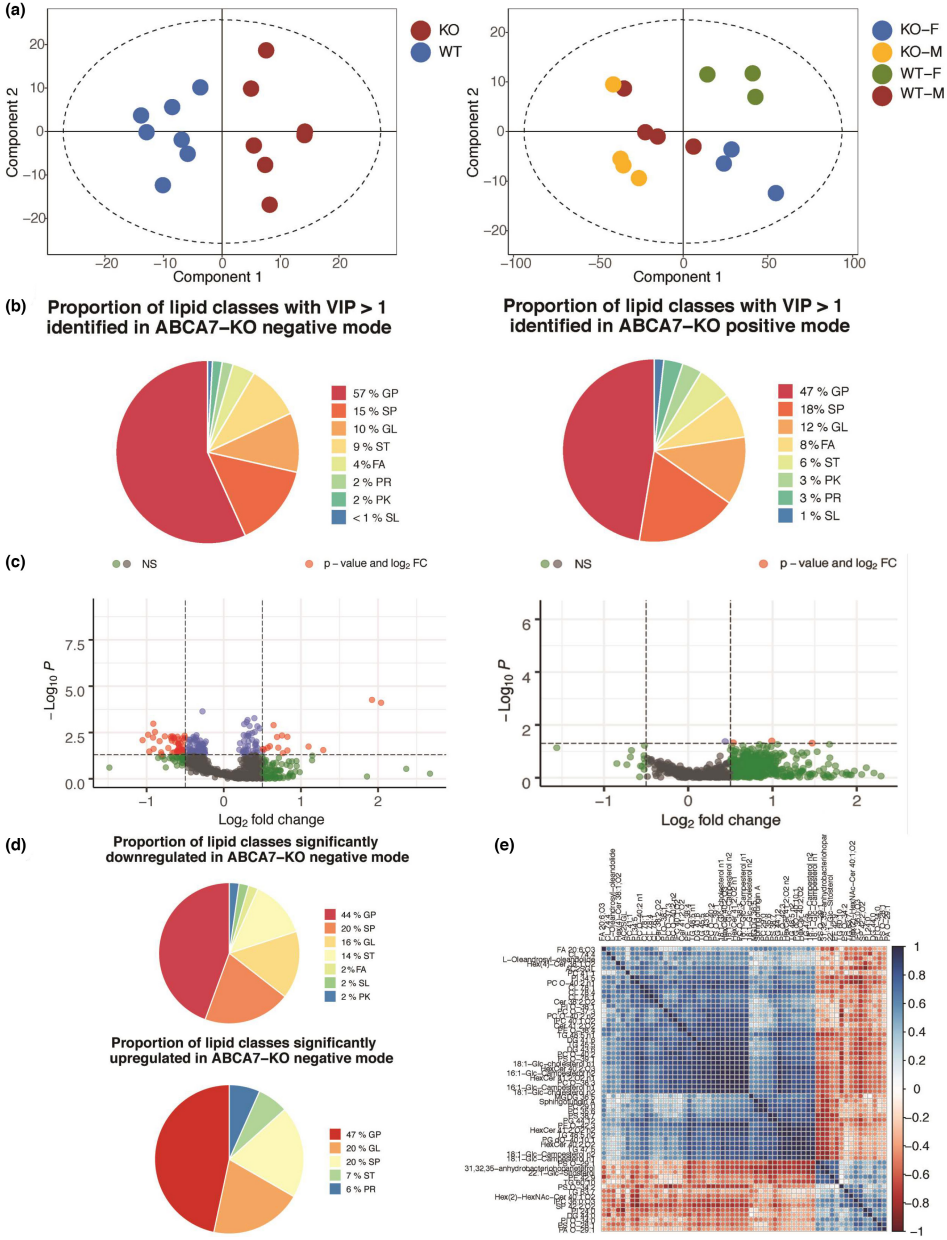
Lipidomic analysis of the ABCA7‐KO mice cortex in the negative and positive modes. (a) OPLS‐DA score plot of the original ABCA7‐KO lipidomics data set in negative mode (R^2^x cum = 0.65, Q^2^ cum = 0.56, R^2^x cum intercept at 0.0, 0.90, and Q^2^ cum intercept at 0.0, −0.35; left) and positive mode (R^2^x cum = 0.93, Q^2^ cum = 0.41, R^2^x cum intercept at 0.0, 0.61, and Q^2^ cum intercept at 0.0, −0.44; right). (b) Proportion of lipid classes with a variable influence of projection (VIP) score above 1 in the negative (left) and positive (right) modes of the ABCA7 KO cortical lipidome. Lipid class abbreviations: FA, fatty acids; GL, glycerolipids; GP, glycerophospholipids; PK, polyketides; PR, prenol lipids; SL, saccharolipid; SP, sphingolipids; ST, sterols. (c) Volcano plot illustrating statistical significance (y‐axis) and relative fold change or rFC (x‐axis) of lipid species in ABCA7‐KO mice compared with WT counterparts in the negative (left) and positive modes (right). Volcano plot individual dots color scheme: Gray and green dots = lipids with non‐significant modulation among ABCA7‐KO and WTs, blue dots = lipids significantly dysregulated in ABCA7‐KO with rFC < ±0.50 (*n* = 115), red dots = lipids significantly dysregulated in ABCA7‐KO with rFC > ±0.50 (*n* = 60). (d) Proportion of lipid classes significantly down‐regulated (up‐) or up‐regulated (down‐) in the negative mode of the ABCA7‐KO cortical lipidome data set compared with WT. Lipid class abbreviations: FA, fatty acids; GL, glycerolipids; GL, glycerolipids; GP, glycerophospholipids; PK, polyketides; PR, prenol lipids; SL, saccharolipid; SP, sphingolipids; ST, sterols. (e) Hierarchical cluster analysis of Spearman correlation patterns among significantly dysregulated lipid species with rFC above ±0.50 in the ABCA7‐KO cortical lipidome in the negative mode.

**FIGURE 6:**
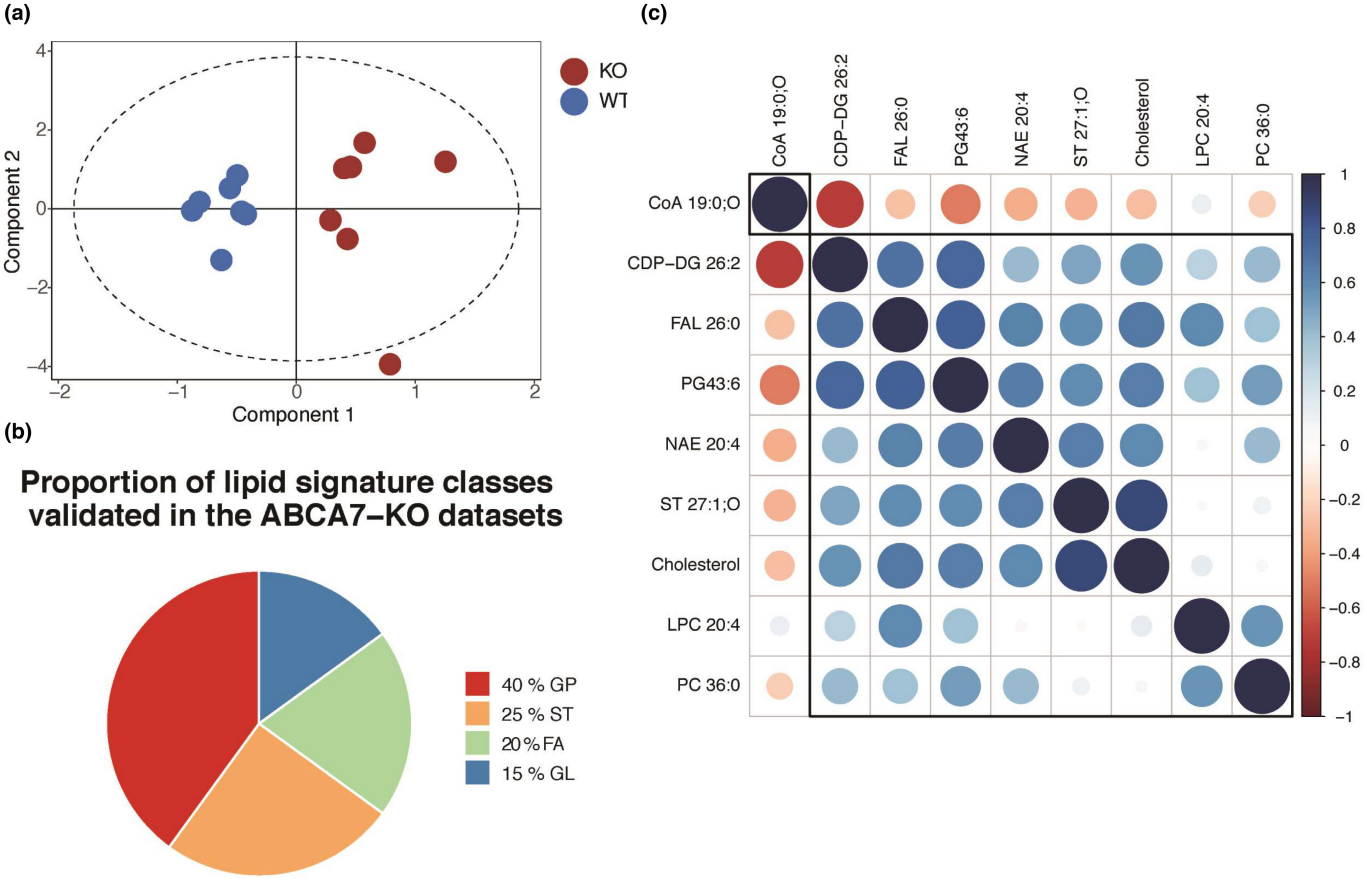
The AD multi‐omics lipid signature is validated in the ABCA7‐KO cortical lipidome (a) OPLS‐DA score plot of the ABCA7 KO lipidomics subset predicted by the AD multi‐omics lipid signature in negative mode (R^2^x cum = 0.91, Q^2^ cum = 0.74, R^2^x cum intercept at 0.0, 1.00, and Q^2^ cum intercept at 0.0, −0.37). (b) Proportion of lipid classes in the ABCA7 KO lipidomics subset predicted by the AD multi‐omics lipid signature in negative and positive modes compared with WT. Lipid class abbreviations: FA, fatty acids; GP, glycerophospholipids, GL, glycerolipids, ST, sterols. (c) Hierarchical cluster analysis of Spearman correlation patterns among the lipid species subset within ABCA7‐KO lipidomics negative mode data set, as predicted by the AD multi‐omics lipid signature.

The OPLS‐DA model for the negative mode‐validated lipid signature was able to separate ABCA7 and WT samples with a higher degree of robustness than the original data set (Q^2^cum = 0.738 and Q^2^cum = 0.56, respectively), which was validated via permutation testing (Table 2 and Figures 5a and 6a).

**TABLE 2:** OPLS‐DA model parameters for each original ABCA7 data set and the validated multi‐omics lipid signatures subsets

Model	Class Number	R^2^x (cum)	R^2^y (cum)	Q^2^ (cum)	100 permutations R^2^y(cum) intercept	100 permutations Q^2^(cum)intercept
Original ABCA7‐KO negative mode	2	0.65	0.90	0.56	(0.0, 0.90)	(0.0, −0.35)
Validated lipid signature, a negative‐mode subset	2	0.91	1.00	0.74	(0.0, 1.00)	(0.0, −0.37)
Original ABCA7‐KO negative mode	4	0.80	0.88	0.36	(0.0, 0.83)	(0.0, −0.27)
Validated lipid signature, a negative‐mode subset	4	0.81	0.77	0.25	(0.0, 0.56)	(0.0, −0.64)
Original ABCA7 KO positive mode	2	0.92	0.80	0.56	(0.0, 0.82)	(0.0, −0.44)
Validated lipid signature, a positive‐mode subset	2	0.94	0.77	0.43	(0.0, 0.57)	(0.0, −0.71)
Original ABCA7 KO positive mode	4	0.93	0.85	0.41	(0.0, 0.61)	(0.0, −0.44)
Validated lipid signature, a positive‐mode subset	4	0.89	0.47	0.20	(0.0, 0.16)	(0.0, −0.31)

Genotype separation was also captured in the OPLS‐DA model for the positive‐mode original data set, although less readily differentiated than its negative‐mode counterpart (Table 2). The robustness of the OPLS‐DA model assessing genotype separation for the positive mode‐validated lipid signature was impacted by the presence of an outlier (Table 2).

A strong genotype–sex interaction influenced sample separation in the original positive‐mode cortical data set (Q^2^cum = 0.406, Figure 6a), but not in the negative‐mode cortical data set (Q^2^cum = 0.358, Table 2). Since the AD multi‐omics data sets did not consider sex composition, the positive mode‐validated lipid signature should not account for genotype–sex interactions either. Indeed, the genotype–sex interaction was not recapitulated in the positive mode‐validated signature subsets (Q^2^cum = 0.20, Table 2), while the same model for the negative subset was not calculated due to the lack of statistical power in the correspondent analysis of the original data set. Therefore, the validated lipid signature in the negative mode seemed robustly influenced by the ABCA7 genotype.

We then inspected the lipid features with VIP scores above 1 in the original ABCA7‐KO data sets to identify lipid species responsible for driving class separation between WT and ABCA7‐KO mice. The class separation between ABCA7‐KO and WTs was driven by glycerophospholipids (57% and 47% of all lipids with VIP above 1), sphingolipids (15% and 18%), glycerolipids (10% and 12%), and sterols (9% and 6%) in negative and positive modes, respectively (Figure 5b). Univariate analysis of all lipid species with a VIP above 1 in the negative mode revealed 175 significant lipid species, of which 60 had a relative fold change (rFC) above ±0.50 (Table S9, Figure 5c). The top 11 differentially expressed lipid species with rFC >0.50 in ABCA7‐KO mice were PI(O‐20:0/18:0), Hex(2)‐KDN‐Cer 42:2;O2, PI(34:5), 31‐hydroxy‐32,35‐anhydrobacteriohopanetetrol, PC(35:6), PG(44:12), asialo‐GM2(d18:1/22:0), PC(P‐40:1), CL(78:1), 18:1‐Glc‐cholesterol, and 16:1‐Glc‐Campesterol (Table S9). Glycerophospholipids were the dominant dysregulated lipid class in the ABCA7‐KO mice, comprising over 44% and 47% of significantly down‐regulated and up‐regulated lipids (Figure 5d). Of note, sterol lipids were proportionally more depleted in the ABCA7‐KO mice compared with their WT counterparts (Figure 5d). Unbiased hierarchical clustering of correlation patterns among significantly modulated lipid species in ABCA7‐KO mice revealed the presence of a major cluster of positively correlated lipids, as well as a smaller yet distinctive cluster of negatively correlated glycerophospholipids (Figure 5e).

In the positive mode, the strong genotype–sex interaction revealed by our multivariate model also influenced univariate analysis, as only 4 lipid species displayed sex‐independent significant differences in the ABCA7‐KO positive mode data set (Table S9, Figure 5c).

We then compared the univariate and correlation pattern results from the global ABCA7‐KO analysis with the validated multi‐omics lipid signature to validate the suitability of our approach. The proportion of significantly modulated lipid classes in the global, ABCA7‐KO negative mode analysis was mirrored in the validated lipid signature, which exhibited a similar proportion of glycerophospholipids (40%) and glycerolipids (15%) (Figure 6b).

Of note, the validated lipid signature exhibited a higher proportion of fatty acids (20%) compared with significantly down‐regulated (2%) and up‐regulated (none) lipid species in the global ABCA7‐KO analysis in the negative mode (Figure 5d).

The AD multi‐omics lipid signature was able to successfully predict significant changes in the ABCA7 cortical lipidome, as observed in Table 3.

**TABLE 3:** Seventeen predicted lipid species in the AD multi‐omics data sets with a VIP score >1 in the ABCA7‐KO cortical lipidome

Predicted lipid species	GSMN's ID	Detected lipids	LIPID MAPS ID	Ionization mode	VIP score	Statistical test
A fatty aldehyde	Fatty‐Aldehydes	FAL 26:0	LMFA06000107	Negative	1.45	0.455
A saturated‐Fatty‐AcylCoA	Saturated Fatty‐acyl CoA	CoA 19:0;O	LMFA07050225	Negative	1.32	0.0530
Lathosterol	CPD‐4186	ST 27:1;O	LMST01010089	Negative	1.19	0.0070^a^
An L‐1‐phosphatidylglycerol	L‐1‐PHOSPHA TIDYLGLYCEROL	PG 43:6	LMGP04010004	Negative	1.14	0.1282
A Phosphatidylcholine	PHOSPHATIDYLCHOLINE	PC 36:0	LMGP01010006	Negative	1.13	0.6200
Cholesterol	CHOLESTEROL	ST 27:1;O	LMST01010001	Negative	1.11	0.0262^a^
A fatty acid	Fatty Acids	NAE 20:4	LMFA08040001	Negative	1.04	0.5350
		FA 18:3	LMFA01030152	Positive	1.09	0.0268^b^
A 1‐acyl glycero‐phosphocholines	1‐Acylglycero‐Phosphocholines	LPC 20:4	LMGP01050140	Negative & Positive	1.03	0.5530
		LPC 18:1	LMGP01050138	Positive	1.17	0.0342^b^
A CDP‐ diacylglycerol	CDPDIACYL‐ GLYCEROL	CDP‐DG 36:2	LMGP13010004	Negative & Positive	1.09	0.0273^b^
A diacylglycerol	DIACYL GLYCEROL	DG 32:0	LMGL02010001	Positive	1.50	0.0291^a^ ^,^ ^b^
		DG 36:0	LMGL02010002	Positive	1.18	0.0227^b^
4α‐hydroxymethyl‐4β‐methyl‐5α‐cholesta‐8,24‐dien‐3β‐ol	CPD‐4575	ST 29:2;O2	LMST01010232	Positive	1.44	0.0291^b^
Ubiquinol‐8	CPD‐9956	Coenzyme Q8	LMPR02010005	Positive	1.07	0.0247^b^
An acyl‐sn‐Glycerol‐3phosphate	ACYL‐SN‐GLYCEROL‐3P	LPA 18:0	LMGP10050005	Positive	1.07	0.0295^b^
7‐dehydro‐cholesterol	CPD‐4187	ST 27:2;O	LMST01010069	Positive	1.04	0.0427^b^

*Note*: The predicted lipid signature was derived from an extracted metabolic subnetwork containing all significantly enriched lipid metabolic pathways in the AD transcriptomics and proteomics data sets, which contained 73 lipid terms. If species in the predicted lipid signatures referred to a lipid class, all the detected compounds belonging to that lipid class were considered for the analysis. This approach yielded 133 unique lipid species, which were mapped to 40 and 20 lipids detected in negative and positive ion modes, respectively. Of these predicted lipid species, 17 had a VIP score >1 in the OPLS‐DA models for the original ABCA7‐KO data sets.

^a^ *p* < 0.050 significance upon unpaired *t* test and Mann–Whitney non‐parametric testing on intensity differences between ABCA7 and WT mice in the original negative‐mode ABCA7‐KO data set.

^b^ Significance upon One‐way ANOVA using B‐H correction for multiple testing on differences between ABCA7 males and ABCA7 females, WT females, or WT males in the original positive‐mode ABCA7 data set.

Furthermore, the major cluster of positively correlated lipids identified in the global ABCA7‐KO univariate analysis was also mirrored by the VIP score >1 validated lipid signature in negative mode, thus supporting the suitability of our approach (Figures 5e & 6c).

### 3.7 Validation of lipid‐AD risk loci associations in the Airwave and RS cohorts

Last, we performed an MWAS using ^1^H NMR spectra of human blood plasma and serum from 3258 individuals from the Airwave and RS cohorts, respectively (Elliott et al., 2014; Ikram et al., 2020). As these cohorts consist of predominantly healthy individuals, we used 47 known AD risk loci to identify AD risk carriers (Jansen et al., 2019; Kunkle et al., 2019; Lambert et al., 2013).

**FIGURE 7:**
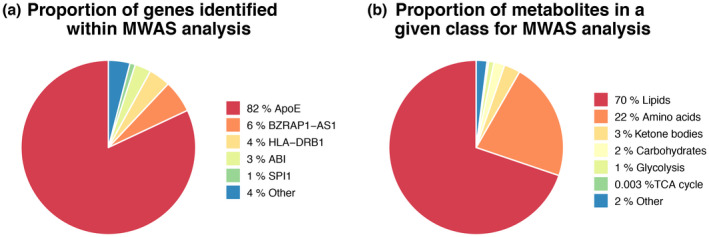
Metabolome‐wide association study of the blood metabolome for AD risk genes in the AIRWAVE and RS cohorts. (a) Proportion of AD risk genes significantly associated with fluctuating metabolite levels detected in the blood samples of individuals in the Airwave and RS cohorts. MWSL was set to 0.050 upon 10 000 permutations to control for FWER. (b) Proportion of metabolite classes associated with AD risk loci in the Airwave and RS cohorts. MWSL was set to 0.050 upon 10 000 permutations to control for FWER.

After performing MWAS, we detected 298 SNP–metabolite associations from the three NMR pulse sequences, out of which 107 were in the lipoprotein, 13 in the CPMG, and 178 in the solvent suppression pulse spectra data sets (Table S10). Association with *APOE* was found for 83% of these, reflecting the importance of this gene in regulating components of the blood metabolome (Figure 7a).

To examine the associations further, we classified the detected metabolites according to their chemical characteristics and biological role into lipids, amino acids, carbohydrates, glycolysis intermediates, TCA cycle intermediates, ketone bodies, and other metabolites. Lipids included resonances that were associated with both protons attached to the fatty acid and the head group (largely choline and glycerol) along with protons from cholesterol and cholesterol esters. The dominant class was represented by lipids, comprising over 70% of the associations (Figure 7b).

## 4 DISCUSSION

The main aim of this study was to validate the presence of metabolic perturbations in AD using multi‐omics pathway‐based integration and metabolic subnetwork extraction. We hypothesized that metabolic alterations detected at multiple omics levels could predict a robust metabolic signature in the AD metabolome. If validated, these results would provide a comprehensive perspective on AD metabolism while supporting the use of GSMNs to identify consistent metabolic alterations in AD.

GO analysis of AD transcriptomics, proteomics, and GWAS‐orthologs data revealed numerous enriched metabolic BP. Although the initial mapping of DE transcripts, proteins, and GWAS‐orthologs certainly removed elements with no metabolic roles, this step did not disproportionately influence metabolic BP term over‐representation per se, as only 3 out of 11 BP in mapped GWAS‐orthologs were metabolic. Lipid and fatty acid BP enrichment were found across the AD all‐mapped transcriptome and proteome. This observation was further supported by *TAL1*, *MAX*, and *BHLHE40* over‐representation in both data sets, as these transcription factors are known lipid and fatty acid metabolism regulators (Carroll et al., 2018; Kassouf et al., 2010).

Pathway and TF enrichment analysis implicated differential metabolic processes across the AD multi‐omics data sets, which also exhibited different cell‐type enrichments. Cholesterol biosynthesis and *SREBF1* were strongly enriched in the AD‐metabolic transcriptome, which was predominantly derived from AD mice modeling amyloid deposition. In agreement with our findings, abnormal levels of cholesterol and its sterol intermediates have been reported in the brain metabolome (Chan et al., 2012) and transcriptome (Zhuang et al., 2019) of AD mice harboring pathogenic mutations in their *APP* and *PS1* loci. The AD‐metabolic transcriptome also exhibited a significant microglia enrichment, thus suggesting microglial cholesterol metabolism might play an important role in AD pathogenesis. These observations are in line with the extensive cholesterol abnormalities reported in TREM2‐defficent microglia, another gene variant heavily implicated in AD pathogenesis (Nugent et al., 2020). Furthermore, cholesterol de novo biosynthesis and catabolism also appear dysregulated in AD post‐mortem brain tissue (Varma et al., 2021), thus suggesting the association between microglia cholesterol dysregulation and AD neuropathology can be effectively modeled in amyloidogenic mice.

Fatty acid biosynthesis, α‐oxidation, and β‐oxidation were also significantly enriched in the predominantly amyloidogenic AD‐metabolic transcriptome, which also exhibited microglia and astrocytic enrichment. These findings are consistent with signs of bioenergetics dysfunction that are commonly found in neurodegeneration (Wang et al., 2020). Recently, defective β‐oxidation in APOE4 astrocytes was found to promote fatty acid accumulation and mitochondrial dysfunction in neurons (Qi et al., 2021), thus supporting a putative lipotoxic role for astrocytic fatty acid metabolism in AD.

Additional signs of mitochondrial dysfunction were found in the AD‐metabolic proteome, which exhibited significant aerobic respiration, TCA cycle, and glycolysis enrichment. Indeed, significant energy metabolism deficits have been detected in human (Johnson et al., 2020) and AD mice brain proteomes modeling both amyloid deposition and tau aggregation (Yu et al., 2018).

The main finding in this study is the validation of a predicted lipid signature derived from an extracted metabolic subnetwork with all significantly enriched lipid pathways in AD multi‐omics data sets. The OPLS‐DA model for the validated lipid signature in negative ion mode LC–MS data set was capable of driving class separation based on ABCA7 genotype with a higher degree of robustness than in the original data set; the reduced number of features was not a confounding factor for the model, but instead allowed for the removal of features originally decreasing the model robustness. Multi‐omics integration is being increasingly used to draw biologically meaningful conclusions over large data sets (Pinu et al., 2019) and has been previously applied to AD data to infer metabolic perturbations using protein ranking and gene‐set enrichment (Bai et al., 2020; Bundy et al., 2019), gene–protein interaction networks (Canchi et al., 2019), and protein–protein interaction networks (Zhang et al., 2020). To our knowledge, this is the first study using multi‐omics pathway‐based integration and metabolic subnetwork extraction to identify and subsequently validate a lipid metabolic signature in the AD lipidome.

Eleven lipid species from the validated lipid signature were significantly modulated in the cortical ABCA7 lipidome, of which four belonged to the cholesterol biosynthesis pathway. Lathosterol and cholesterol were significantly decreased in the ABCA7‐KO lipidome compared with WT, whereas 7‐dehydro‐cholesterol and 4α‐hydroxymethyl‐4β‐methyl‐5α‐cholesta‐8,24‐dien‐3β‐ol were significantly decreased in ABCA7‐females compared with ABCA7 males. The evidence is mixed regarding cholesterol and intermediate sterols changes in ABCA7 mice. One study showed no cholesterol changes in ABCA7‐KO mice brains (Satoh et al., 2015); serum cholesterol levels were, however, decreased in female ABCA7‐KO mice (Kim et al., 2005). This study appears more aligned with the latter, as decreased free‐cholesterol levels and sex‐specific sterol intermediates differences were detected. This discrepancy is extended to other AD mouse models. Free‐cholesterol and lathosterol levels exhibited non‐significant changes in TgCRND8 (Yang et al., 2014) and APP/PS1 mice (Bogie et al., 2019), whereas lanosterol and cholesteryl acetate were up‐regulated in APOE4 mice (Nuriel et al., 2017). Despite these disagreements, the importance of sterol intermediates in AD is reflected therapeutically, as a recent drug‐repurposing screen identified several tau‐reducing compounds which targeted cholesterol esters (van der Kant et al., 2019).

We also performed an MWAS analysis using SNPs previously associated with LOAD and metabolites detected in blood plasma and serum from the Airwave and Rotterdam cohorts, respectively, using ^1^H NMR spectroscopy. Mean ages of recruitment in these cohorts are relatively young, and thus our reported 298 SNP‐metabolite associations may represent early stages of the disease, as the brain begins to accumulate neurodegenerative features that ultimately result in mild cognitive impairment (MCI) and AD. Using three distinct NMR pulse sequences, we were able to detect a range of metabolites including lipids, amino acids, glycolysis, TCA cycle intermediates, and ketone bodies. Lipids were the commonest metabolite class represented in metabolite‐SNP associations, suggesting that dysregulation of lipid metabolism may be some of the earliest events in AD.

There are important limitations associated with this study. First, this study included multi‐omics data from several brain regions, ages, and AD mouse models, and therefore region and age‐specific metabolic alterations that are frequent in AD (González‐Domínguez et al., 2014) were not assessed. It is also notoriously difficult to annotate lipid species into GSMNs owing to the complexities associated with lipid nomenclature and identification (Poupin et al., 2020). Given its dry‐lab nature, this study had to rely on exact mass and database‐based level 2 and level 3 annotations to assign GSMN‐extracted annotations to individual lipid species, as established in consensus reporting standards for metabolite identification (Salek et al., 2013). As a result, we had to resort to the use of lipid classes and second‐order annotations rather than identifications. Such an approach though could be used as a part of the metabolite identification workflow in open profiling metabolomics and lipidomics.

By doing so, this study employed lipid classes and second‐order annotations rather than exact identifications in the predicted AD multi‐omics lipid signature. Despite its limitations, this approach is currently accepted in lipid annotation workflows in open profiling lipidomics (Poupin et al., 2020). This study was also limited in that cell‐type enrichment analysis could not distinguish whether astrocytic and microglia enrichment was associated with gliosis in disease rather than AD pathology per se, as cell‐type proportions could not be adequately controlled in silico.

Additionally, APOE‐associated SNPs dominated our MWAS analysis, which could be attributed to the known association of ApoE with dyslipidemia and atherosclerosis (Bouchareychas & Raffai, 2018). Furthermore, ^1^H NMR spectra of blood plasma detect a high proportion of lipids compared with other classes of metabolites and are relatively insensitive as a technique. We are currently performing mass spectrometry to expand the coverage of the metabolome to further investigate the earliest molecular events in AD.

Last, our use of lipidomics data from cortical AD mice and human AD blood extracts has distinct implications. By including data sets from AD animal models, our findings describe metabolic abnormalities underlying familiar AD, whereas, in AD patients, the disease is mostly sporadic in origin. This study minimized this limitation in the validation stage by performing an MWAS using only metabolic profiles from individuals with known AD risk SNPs, therefore reducing the proportion of sporadic AD in our human cohorts. Additionally, direct comparisons between the blood and brain lipidome should be exercised with caution, as they provide insights into different disease aspects. The blood metabolome reflects systemic metabolic patterns in AD patients, whereas dysregulated analytes from the brain lipidome are spatially related to neuropathological insults, and therefore might provide direct insights into disease etiology (Jové et al., 2014).

## 5 CONCLUSIONS

In summary, this study highlights the suitability of integrating multi‐omics data into GSMNs to identify metabolic alterations in AD. Pathway‐based integration of multi‐omics data revealed distinct perturbations in lipid metabolism in the AD mouse brain. Predicted lipids extracted from the over‐represented lipid pathway's metabolic subnetwork were validated in the ABCA7 lipidome, with its associated multivariate model robustly modeling class separation. Furthermore, more than 70% of 298 SNP‐metabolite associations in an MWAS corresponded to lipid species, thus validating the presence of lipidomic dysregulation in AD.

## AUTHOR CONTRIBUTIONS

M.E.G.S. and J.L.G. conceived and designed the study. M.E.G.S. retrieved and analyzed the transcriptomics, proteomics, GWAS, and lipidomics data. B.R.D. acquired the lipidomics data. S.L. and BRD processed the lipidomics data. I.K. performed the MWAS study, which used data from two ongoing cohorts overseen by P.E. M.E.G.S. and J.L.G. interpreted the data. M.E.G.S. drafted the manuscript, which received critical input from J.L.G and S.L. All authors have read and approved the published version of the manuscript.

## CONFLICT OF INTEREST

The authors declare that they have no conflict of interest.

## Supporting information


Data S1

Table S1

Table S2

Table S8

Table S9

Table S10

## Data Availability

The manuscript makes secondary use of data already available through public databases. We have listed all the datasets we have accessed allowing others to reproduce our analysis. A preprint of this article has been posted on MedRxiv on May 17, 2021: https://www.medrxiv.org/content/10.1101/2021.05.10.21255052v1.
