# Supplementary material for: Pathway‐based integration of multi‐omics data reveals lipidomics alterations validated in an Alzheimer's disease mouse model and risk loci carriers
Source: J Neurochem. 2022 Dec 12;164(1):57–76. doi: 10.1111/jnc.15719 (PMC10107183; doi:10.1111/jnc.15719)
Supplement: Supplementary file 1 — Data S1 [file JNC-164-57-s001.pdf]

Supplementary Data for Pathway-based integration of multi-omics data reveals lipidomics alterations validated in an Alzheimer´s Disease mouse model and risk loci carriers

Monica Emili Garcia-Segura[1,2], Brenan R. Durainayagam[2,3], Sonia Liggi[2], Gonçalo Graça[4], Beatriz Jimenez[5], Abbas Dehghan[3,6,7], Ioanna Tzoulaki[3,6,8], Ibrahim Karaman[4,6], Paul Elliott[3,6,7,8] and Julian L. Griffin[2,3,10, 11]*

[1] Department of Brain Sciences, Imperial College London, London, UK.
[2] Section of Biomolecular Medicine, Department of Metabolism, Digestion and Reproduction, Imperial College London, London, UK.
[3] UK-Dementia Research Institute (UK-DRI) at Imperial College London, London, UK.
[4] Section of Bioinformatics, Department of Metabolism, Digestion and Reproduction, Imperial College London, London, UK.
[5] Section of Bioanalytical Chemistry and the National Phenome Centre, Department of Metabolism, Digestion and Reproduction, Imperial College London, London, UK.
[6] Department of Epidemiology and Biostatistics, Imperial College London, London, UK.
[7] MRC Centre for Environment and Health, Imperial College London, London, UK.
[8] National Institute for Health Research Imperial Biomedical Research Centre, Imperial College London, UK.
[9] Department of Hygene and Epidemiology, University of Ioannina Medical School, University Campus Road 455 00, Ioannina, Greece.
[10] Department of Biochemistry and Cambridge Systems Biology Centre, University of Cambridge, Cambridge, UK.
[11] The Rowett Institute, University of Aberdeen, Aberdeen, Scotland, UK.

Correspondence
Julian L. Griffin, Biomolecular Medicine, Division of Systems Medicine, Department of Metabolism, Digestion and Reproduction, Imperial College London, London, United Kingdom. Email: julian.griffin@imperial.ac.uk ; Tel.: +44-(0)20-7594-3220

Supplementary Table 1: Top-20 differentially expressed (DE) transcripts or proteins in each dataset (based on q-value) employed in this study. Please see Excel spread sheet Supplementary Table 1.

Supplementary table 2: DE transcripts/proteins/GWAS orthologs successfully mapped to the genome-scale metabolic network (referred in the text as all-mapped AD multi-omics elements). Please see Excel spread sheet Supplementary Table 2.

Supplementary Table 3. Transcription Factor enrichment analysis of all-mapped AD transcriptomics and proteomics datasets

Transcription factor	Gene or protein target coverage	Fishers exact test p-value	FDR adj. p-value	Odds Ratio
**Transcriptomics**	**all**			
CTCF	83/1646	4.28e-07	2.36e-04	1.906
MAFK	76/1616	1.20e-50	0.0013	1.772
MAZ	78/1766	6.92e-05	0.0029	1.659
CEBPB	60/1285	9.48e-05	0.0035	1.758
ETS1	71/1614	1.48e-04	0.0051	1.652
MAX	113/2892	3.24e-04	0.0089	1.46
EGR1	73/1773	7.25e-04	0.016	1.542
TAL1	77/1904	8.77e-04	0.019	1.513
MYOD1	40/860	0.0011	0.023	1.751
BHLHE40	70/1727	0.0013	0.025	1.517
TCF3	69/1708	0.0015	0.026	1.511
NFYA	73/1834	0.0017	0.028	1.488
TBP	68/1687	0.0017	0.028	1.508
USF2	71/1814	0.0027	0.032	1.462
HNF4A	89/2396	0.0040	0.042	1.385
NR3C1	13/204	0.0043	0.043	2.443
JUN	53/1325	0.0053	0.048	1.496
**Proteomics**	**All**			
TAL1	35/690	5.41e-07	4.97e-05	2.759
SREBF1	23/455	3.97e-05	8.36e-04	2.749
SREBF2	15/366	0.0055	0.017	2.206
BHLHE40	67/1702	2.12e-07	2.82e-05	2.116
FOS	45/1146	1.43e-05	4.66e-04	2.11
SP4	53/1367	4.33e-06	1.99e-04	2.083
REST	70/1816	2.56e-07	2.82e-05	2.07
MAX	65/1728	1.31e-06	7.25e-05	2.018
MXI1	66/1760	1.23e-06	7.25e-05	2.012
CTCF	68/1821	1.03e-06	7.25e-05	2.003
NRF1	68/1827	1.14e-06	7.25e-05	1.996
MYC	64/1726	2.40e-06	1.21e-04	1.988
MAFK	43/1169	8.83e-05	0.0010	1.972
ESRRA	34/934	4.88e-04	0.0030	1.951
MAZ	62/1714	6.80e-06	2.89e-04	1.938
CEBPB	64/1836	1.49e-05	4.66e-04	1.865
IRF3	58/1688	4.80e-05	8.36e-04	1.837
ZBTB7A	59/1735	5.52e-05	8.47e-04	1.818
TBP	62/1833	4.40e-05	8.36e-04	1.808
CREB1	58/1731	9.07e-05	0.00104	1.79
ETS1	54/1614	1.52e-04	0.0015	1.787
EGR1	54/1627	1.83e-04	0.0015	1.772
PAX5	27/817	0.0058	0.017	1.765
E2F4	61/1847	9.22e-05	0.0010	1.763
ATF3	42/1278	9.37e-04	0.0047	1.754
USF1	81/2480	1.52e-05	4.66e-04	1.743
RELA	59/1815	1.71e-04	0.0015	1.735
USF2	59/1820	1.82e-04	0.0015	1.73
NFYB	60/1852	1.66e-04	0.0015	1.729
ZKSCAN1	60/1857	1.78e-04	0.0015	1.724
NFYA	59/1834	2.19e-04	0.0017	1.716
BACH1	58/1814	2.81e-04	0.0020	1.705
RFX5	58/1832	3.54e-04	0.0024	1.688
YY1	84/2657	3.24e-05	0.00075	1.686
TCF3	54/1708	5.43e-04	0.0032	1.686

STAT1	58/1855	4.70e-04	0.0029	1.666
TFAP2C	56/1822	8.25e-04	0.0043	1.637
SRF	45/1467	0.0023	0.0088	1.634
NR2C2	41/1343	0.0037	0.013	1.626
NFE2	56/1854	0.0012	0.0057	1.608
MYOD1	84/2784	1.35e-04	0.0013	1.606
GATA3	30/997	0.013	0.030	1.602
TCF12	30/1012	0.015	0.035	1.577
PBX3	58/1958	0.0015	0.0068	1.576
MYBL2	44/1487	0.0047	0.015	1.574
STAT5A	36/1230	0.011	0.026	1.557
E2F6	71/2432	8.27e-04	0.0043	1.553
SP1	47/1611	0.0047	0.015	1.552
SP2	60/2063	0.0019	0.0077	1.547
ARID3A	53/1828	0.0034	0.012	1.542
TEAD4	36/1245	0.013	0.029	1.537
CTCFL	62/2154	0.0021	0.0081	1.53
JUND	52/1821	0.0048	0.015	1.518
EBF1	75/2676	0.0017	0.0073	1.489
ELK1	51/1837	0.0084	0.022	1.474
ELF1	52/1875	0.0080	0.022	1.473
TFAP2A	50/1810	0.0096	0.024	1.467
POU2F2	43/1563	0.0156	0.035	1.461
ATF1	50/1833	0.012	0.028	1.448
GABPA	62/2285	0.0068	0.019	1.44
E2F1	50/1854	0.014	0.032	1.431
TCF7L2	69/2574	0.0062	0.018	1.422
HMGN3	49/1830	0.0167	0.036	1.421
CUX1	48/1827	0.023	0.047	1.393
IRF1	105/4204	0.0083	0.022	1.323
STAT3	85/3450	0.019	0.040	1.304

FDR adj. refers to Benjamini-Hochberg (B-H) False Discovery Rate correction for multiple testing.

Supplementary Table 4. Biological Process (BP), Molecular Function (MF) and Cellular Compartment (CC) enrichment analysis of all-mapped AD transcriptomics, proteomics and GWAS-orthologs datasets

GO ID	Term	Term Coverage (mapped/all)	Fold Enrichment	P value	FDR adj. p value
	**Transcriptomics (BP)**				
GO:0016310	Phosphorylation	105/612	5.36	1.32e-46	2.34e-43
GO:0006468	Protein phosphorylation	90/576	4.88	2.19e-36	3.88e-33
GO:0055114	Oxidation-reduction process	95/676	4.39	1.23e-34	2.18e-31
GO:0008152	Metabolic process	74/463	4.99	2.01e-30	3.57e-27
GO:0006629	Lipid metabolic process	72/459	4.90	4.54e-29	8.05e-26
GO:0046777	Protein autophosphorylation	46/183	7.85	2.15e-27	3.81e-24
GO:0035556	Intracellular signal transduction	51/400	3.98	9.65e-17	2.00e-13
GO:0006631	Fatty acid metabolic process	30/156	6.00	1.36e-14	2.42e-11
GO:0018105	Peptidyl-serine phosphorylation	26/133	6.10	7.13e-13	1.27e-09
GO:0018108	Peptidyl-tyrosine phosphorylation	18/65	8.65	1.35e-11	2.40e-08
GO:0005975	Carbohydrate metabolic process	28/206	4.24	5.09e-10	9.03e-07
GO:0006198	cAMP catabolic process	9/16	17.56	1.07e-8	1.89e-05
GO:0006811	Ion transport	47/568	2.51	1.77e-08	3.13e-05
GO:0038083	Peptidyl-tyrosine autophosphorylation	12/41	9.14	4.28e-08	7.60e-05
GO:0006470	Protein dephosphorylation	20/138	4.53	8.56e-08	1.52e-04
GO:0016126	Sterol biosynthetic process	10/27	11.566	9.17e-08	1.63e-04
GO:0006694	Steroid biosynthetic process	13/63	6.55	5.10e-07	9,05e-4
GO:0007169	Transmembrane receptor protein tyrosine kinase signaling pathway	16/100	4.99	6.40e-07	0.0011
GO:0016042	Lipid catabolic process	16/109	4.58	1.97e-06	0.0035
GO:0016311	Dephosphorylation	16/109	4.58	1.97e-06	0.0035
GO:0055085	Transmembrane transport	31/364	2.66	2.28e-06	0.0040
GO:0006749	Glutathione metabolic process	11/49	7.01	2.75e-06	0.0048
GO:0006695	Cholesterol biosynthetic process	9/32	8.78	5.54e-06	0.009
GO:0010033	Response to organic substance	12/65	5.76	6.05e-06	0.010
GO:0034765	Regulation of ion transmembrane transport	17/135	3.93	6.61e-06	0.011
GO:0006635	Fatty acid beta-oxidation	10/44	7.09	8.54e-06	0.015
GO:0042493	Response to drug	28/339	2.57	1.36e-05	0.024
GO:0008202	Steroid metabolic process	13/84	4.83	1.41e-05	0.025
GO:0009636	Response to toxic substance	13/86	4.72	1.80e-05	0.032
GO:0006486	Protein glycosylation	15/116	4.04	2.01e-05	0.035
	**Proteomics (BP)**				
GO:0055114	Oxidation-reduction process	74/676	5.08	4.82e-31	8.27e-28
GO:0008152	Metabolic process	59/463	5.90	5.11e-28	8.77e-25
GO:0016310	Phosphorylation	57/612	4.31	2.32e-20	3.98e-17
GO:0006629	Lipid metabolic process	44/459	4.44	4.54e-16	7.66e-13
GO:0006468	Protein phosphorylation	46/576	3.70	6.68e-14	1.14e-10
GO:0006099	Tricarboxylic acid cycle	12/29	19.18	9.70e-12	1.66e-08
GO:0008652	Cellular amino-acid biosynthetic process	9/25	16.69	3.35e-08	5.75e-05
GO:0006631	Fatty acid metabolic process	18/156	5.35	4.57e-08	7.85e-05
GO:0005975	Carbohydrate metabolic process	20/206	4.50	1.10e-07	1.89e-04
GO:0045454	Cell redox homeostasis	12/65	8.56	1.25e-07	2.15e-04
GO:0006470	Protein dephosphorylation	16/138	5.37	2.94e-07	5.05e-04
GO:0006749	Glutathione metabolic process	10/49	9.46	8.64e-07	0.0015
GO:0035556	Intracellular signal transduction	25/400	2.89	6.45e-06	0.011
GO:0046777	Protein autophosphorylation	16/183	4.05	1.03e-05	0.017
GO:0009058	Biosynthetic process	8/37	10.02	1.19e-05	0.021
GO:0006006	Glucose metabolic process	10/68	6.81	1.45e-05	0.025
GO:0016311	Dephosphorylation	12/109	5.10	2.31e-05	0.039
	**GWAS-orthologs (BP)**				
GO:0016310	Phosphorylation	50/612	5.75	2.16e-23	3.56e-20
GO:0006468	Protein phosphorylation	44/576	5.37	2.08e-19	3.43e-06

GO:0046777	Protein autophosphorylation	19/183	7.30	1.25e-10	2.07e-07
GO:0006811	Ion transport	27/584	3.25	2.63e-07	4.35e-04
GO:0006470	Protein dephosphorylation	13/138	6.63	6.30e-07	0.0010
GO:0008152	Metabolic process	23/463	3.50	7.86e-07	0.0013
GO:0055085	Transmembrane transport	20/364	3.86	1.15e-06	0.0019
GO:0034765	Ion transmembrane transport regulation	12/135	6.25	3.52e-06	0.0058
GO:0055114	Oxidation-reduction process	27/676	2.81	4.05e-06	0.0067
GO:0006629	Lipid metabolic process	21/459	3.21	9.22e-06	0.015
GO:0018105	Peptidyl-serine phosphorylation	11/133	5.82	1.99e-05	0.033
	**Transcriptomics (MF)**				
GO:0016740	Transferase activity	191/1472	3.84	4.04e-64	6.42e-61
GO:0016301	Kinase activity	109/674	4.79	9.91e-44	1.58e-40
GO:0004672	Protein kinase activity	83/531	4.63	7.05e-32	1.12e-28
GO:0016491	Oxidoreductase activity	88/604	4.31	1.63e-31	2.60e-28
GO:0005524	ATP binding	142/1507	2.79	2.60e-30	4.14e-27
GO:0000166	Nucleotide binding	157/1936	2.40	1.66e-26	2.63e-23
GO:0016787	Hydrolase activity	136/1533	2.62	2.43e-26	3.88e-23
GO:0003824	Catalytic activity	69/479	4.27	2.50e-24	3.98e-21
GO:0004674	Protein serine/threonine kinase activity	63/428	4.36	1.03e-22	1.64e-19
GO:0004713	Protein tyrosine kinase activity	26/121	6.36	2.40e-13	3.81e-10
GO:0008081	Phosphoric diester hydrolase activity	15/52	8.54	1.14e-09	1.81e-06
GO:0051287	NAD binding	15/55	8.08	2.55e-09	4.05e-06
GO:0004721	Phosphoprotein phosphatase activity	22/139	4.67	8.29e-09	1.32e-05
GO:0016746	Transferase activity, transferring acyl groups	24/167	4.26	1.00e-08	1.59e-05
GO:0004715	Non-membrane spanning prot. tyrosine kinase act.	13/45	8.55	2.00e-08	3.18e-05
GO:0016829	Lyase activity	21/139	4.48	4.37e-08	6.95e-04
GO:0004029	Aldehyde dehydrogenase (NAD) activity	9/19	14.03	8.64e-08	1.38e-04
GO:0016757	Transferase activity, transf. glycosyl groups	25/208	3.56	1.54e-07	2.45e-04
GO:0016791	Phosphatase activity	18/118	4.52	4.40e-07	7.00e-04
GO:0046872	Metal ion binding	162/3355	1.43	6.58e-07	0.0010
GO:0004114	3',5'-cyclic-nucleotide phosphodiesterase activity	9/24	11.11	7.24e-07	0.0012
GO:0004725	Protein tyrosine phosphatase activity	16/97	4.88	8.29e-07	0.0013
GO:0005536	Glucose binding	7/12	17.38	1.11e-06	0.0018
GO:0008234	Cysteine-type peptidase activity	19/141	3.99	1.26e-06	0.0020
GO:0004714	Transmembrane recept. protein tyrosine kinase act.	12/54	6.58	1.49e-06	0.0023
GO:0004115	3',5'-cyclic-AMP phosphodiesterase activity	7/13	15.94	2.00e-06	0.0032
GO:0004602	Glutathione peroxidase activity	8/20	11.85	2.52e-06	0.0040
GO:0015293	Symporter activity	16/112	4.23	5.28e-06	0.0084
GO:0016853	Isomerase activity	16/115	4.12	7.34e-06	0.011
GO:0016620	Oxidoreductase activity, acting on the aldehyde or oxo group of donors	9/32	8.33	8.16e-06	0.013
GO:0004364	Glutathione transferase activity	9/33	8.08	1.05e-05	0.016
GO:0005244	Voltage-gated ion channel activity	17/134	3.76	1.16e-05	0.018
GO:0000287	Magnesium ion binding	21/202	3.08	1.70e-05	0.027
GO:0030552	cAMP binding	8/26	9.11	1.79e-05	0.028
	**Proteomics (MF)**				
GO:0016740	Transferase activity	105/1472	3.25	4.27e-28	6.59e-25
GO:0003824	Catalytic activity	60/479	5.71	9.72e-28	1.50e-24
GO:0016787	Hydrolase activity	99/1533	2.94	4.30e-23	6.64e-20
GO:0005524	ATP binding	98/1507	2.96	4.63e-23	7.26e-20
GO:0000166	Nucleotide binding	111/1936	2.61	5.49e-22	8.49e-19
GO:0016491	Oxidoreductase activity	59/604	4.45	9.33e-22	1.44e-18
GO:0016301	Kinase activity	61/674	4.12	7.73e-21	1.19e-17
GO:0004672	Protein kinase activity	43/531	3.67	5.61e-13	8.66e-10
GO:0004674	Protein serine/threonine kinase activity	36/428	3.83	2.13e-11	3.29e-08
GO:0051287	NAD binding	12/55	9.94	2.42e-08	3.74e-05
GO:0016853	Isomerase activity	16/115	6.33	3.18e-08	4.91e-05
GO:0004702	Receptor sig. protein serine/threonine kinase act.	12/57	9.58	3.59e-08	5.55e-05
GO:0004721	Phosphoprotein phosphatase activity	17/139	5.57	6.66e-08	1.03e-04

GO:0016829	Lyase activity	17/139	5.57	6.66e-08	1.03e-04
GO:0008081	Phosphoric diester hydrolase activity	11/52	9.63	1.57e-07	2.43e-04
GO:0000287	Magnesium ion binding	18/202	4.06	2.34e-06	0.0036
GO:0042803	Protein homodimerization activity	40/798	2.28	2.42e-06	0.0037
GO:0030170	Pyridoxal phosphate binding	10/55	8.28	2.75e-06	0.0042
GO:0019901	Protein kinase binding	27/434	2.83	3.81e-06	0.0059
GO:0004725	Protein tyrosine phosphatase activity	12/97	5.63	8.86e-06	0.014
GO:0008137	NADH dehydrogenase (ubiquinone) activity	8/35	10.41	9.05e-06	0.014
GO:0016791	Phosphatase activity	13/118	5.01	1.08e-05	0.017
GO:0019899	Enzyme binding	24/384	2.84	1.39e-05	0.021
GO:0016874	Ligase activity	23/362	2.89	1.71e-05	0.026
GO:0009055	Electron carrier activity	9/53	7.74	1.84e-05	0.028
	**GWAS-orthologs (MF)**				
GO:0016740	Transferase activity	93/1472	4.30	1.94e-35	2.87e-32
GO:0016301	Kinase activity	52/674	5.25	1.22e-22	1.80e-19
GO:0005524	ATP binding	74/1507	3.34	7.27e-21	1.07e-17
GO:0004672	Protein kinase activity	42/531	5.40	1.40e-18	2.07e-15
GO:0000166	Nucleotide binding	77/1936	2.71	2.73e-16	3.33e-13
GO:0004674	Protein serine/threonine kinase activity	34/428	5.41	5.10e-15	7.54e-12
GO:0016829	Lyase activity	16/139	7.84	1.91e-09	2.83e-06
GO:0003824	Catalytic activity	28/479	3.98	2.16e-09	3.18e-06
GO:0046872	Metal ion binding	88/3355	1.78	1.16e-08	1.71e-05
GO:0004721	Phosphoprotein phosphatase activity	14/139	6.86	1.30e-07	1.92e-04
GO:0016791	Phosphatase activity	12/118	6.93	1.26e-06	0..0019
GO:0000287	Magnesium ion binding	15/202	5.06	1.69e-06	0.0025
GO:0005244	Voltage-gated ion channel activity	12/134	6.10	4.43e-06	0.0065
GO:0016853	Isomerase activity	11/115	6.51	7.24e-06	0.011
GO:0016491	Oxidoreductase activity	25/604	2.82	9.43e-06	0.013
GO:0004812	Aminoacyl-tRNA ligase activity	7/39	12.23	2.00e-05	0.029
GO:0016787	Hydrolase activity	44/1533	1.95	2.41e-05	0.035
	**Transcriptomics (CC)**				
GO:0005829	Cytosol	136/1784	2.63	2.11e-26	2.92e-23
GO:0070062	Extracellular exosome	167/2674	2.15	3.07e-23	4.26e-20
GO:0016020	Membrane	313/6998	1.54	6.74e-22	9.35e-19
GO:0005739	Mitochondrion	121/1721	2.43	2.22e-20	3.09e-17
GO:0005737	Cytoplasm	266/6631	1.39	6.73e-11	9.34e-08
GO:0005783	Endoplasmic reticulum	81/1323	2.11	2.23e-10	3.10e-07
GO:0005764	Lysosome	33/331	3.44	2.60e-09	3.61e-06
GO:0005759	Mitochondrial matrix	22/188	4.04	1.29e-07	1.79e-04
GO:0045121	Membrane raft	26/262	3.43	1.87e-07	2.60e-04
GO:0005743	Mitochondrial inner membrane	32/387	2.86	3.43e-07	4.77e-04
GO:0005789	Endoplasmic reticulum membrane	45/710	2.19	1.80e-06	0.0025
GO:0016323	Basolateral plasma membrane	21/203	3.57	1.92e-06	0.0026
GO:0005794	Golgi apparatus	64/1190	1.86	2.39e-06	0.0033
GO:0043025	Neuronal cell body	37/534	2.39	2.45e-06	0.0034
GO:0031234	Extrinsic – plasma membrane cytoplasmic side	12/68	6.09	3.66e-06	0.0051
GO:0043005	Neuron projection	31/420	2.55	5.52e-06	0.0076
GO:0043231	Intracellular membrane-bounded organelle	45/751	2.07	7.56e-06	0.010
GO:0048471	Perinuclear region of cytoplasm	41/692	2.04	2.70e-05	0.037
GO:0005887	Plasma membrane	58/1126	1.77	2.71e-05	0.037
GO:0043204	Perikaryon	16/250	3.68	3.04e-05	0.042
GO:0016324	Apical plasma membrane	25/328	2.63	3.23e-05	0.045
	**Proteomics (CC)**				
GO:0005739	Mitochondrion	147/1721	4.36	4.68e-57	6.33e-54
GO:0070062	Extracellular exosome	146/2674	2.78	3.45e-33	4.66e-30
GO:0005743	Mitochondrial inner membrane	43/387	5.67	1.24e-19	1.67e-16
GO:0005829	Cytosol	93/1784	2.66	1.30e-18	1.76e-15
GO:0005737	Cytoplasm	206/6631	1.58	1.06e-15	1.50e-12
GO:0005747	Mitochondrial res. chain complex I	16/47	17.38	7.49e-15	1.00e-11

GO:0070469	Respiratory chain	17/58	14.96	1.24e-14	1.68e-11
GO:0043209	Myelin sheath	24/192	6.38	3.99e-12	5.39e-09
GO:0005759	Mitochondrial matrix	23/188	6.24	1.89e-11	2.55e-08
GO:0016020	Membrane	195/6998	1.43	1.06e-09	1.43e-06
GO:0005777	Peroxisome	15/131	5.85	2.87e-07	3.88e-04
GO:0022624	Proteasome accessory complex	7/17	21.02	5.51e-07	7.44e-04
GO:0043231	Intracellular membrane-bounded organelle	37/751	2.51	7.21e-07	9.74e-04
GO:0043005	Neuron projection	25/420	3.04	3.00e-06	0.0041
GO:0008540	Proteasome regulatory particle I	6/13	23.57	3.13e-06	0.0040
GO:0000502	Proteasome complex	10/66	7.73	5.22e-06	0.0070
GO:0005891	Voltage-gated calcium channel complex	7/24	14.89	5.33e-06	0.0072
GO:0005778	Peroxisomal membrane	9/23	8.67	8.09e-06	0.011
GO:0005838	Proteasome regulatory particle II	5/10	25.53	2.74e-05	0.037
GO:0031597	Cytosolic proteasome complex	5/10	25.53	2.74e-05	0.037
GO:0005749	Mitochondrial respiratory chain complex I	4/4	51.07	2.91e-05	0.039
	**GWAS-orthologs (CC)**				
GO:0016020	Membrane	136/6998	1.51	3.42e-09	4.45e-06

FDR adj. refers to Benjamini-Hochberg (B-H) False Discovery Rate correction for multiple testing.

Supplementary Table 5. Transcription Factor enrichment analysis of AD-metabolic transcriptomics and proteomics datasets

Transcription factor	Gene or protein target coverage	Fishers exact test p-value	FDR adj. p-value	Odds Ratio
**AD-metabolic**	**Transcriptomics**			
ESRRA	8/108	1.16e-05	0.00321	8.341
SREBF1	17/455	4.15e-06	0.00229	4.047
HNF4G	16/556	1.62e-04	0.0297	3.089
TAL1	19/820	6.34e-04	0.05	2.473
CTCF	38/2137	6.05e-3	0.05	1.888
MAX	49/2892	3.90e-4	0.05	1.797
**AD-metabolic**	**Proteomics**			
ESRRA	7/108	3.31e-05	0.0013	8.594
NFE2	10/258	6,72e-05	0.0019	5
SREBF1	14/455	3.65e-05	0.0013	3.937
SP4	32/1367	5.77e-07	2.95e-04	2.972
TAL1	16/690	2.81e-04	0.0039	2.944
CEBPB	24/1083	2.57e-05	0.0013	2.81
SREBF2	8/366	0.011	0.037	2.771
MAX	36/1719	1.65e-06	3.03e-04	2.652
JUND	12/598	0.0045	0.020	2.539
CREB1	20/1013	4.45e-04	0.0047	2.497
ATF3	25/1278	1.22e-04	0.0029	2.474
NRF1	35/1827	1.33e-05	0.0011	2.422
USF1	47/2480	1.07e-06	2.95e-04	2.395
USF2	34/1820	2.72e-05	0.0013	2.361
MAZ	32/1714	4.40e-05	0.0014	2.359
MYC	34/1830	3.02e-05	0.0013	2.347
BHLHE40	31/1702	8.50e-05	0.0022	2.3
NR2C2	24/1343	5.57e-04	0.0053	2.256
MXI1	31/1760	1.49e-04	0.0032	2.223
CTCF	32/1825	1.30e-04	0.003	2.213
EGR1	31/1773	1.68e-04	0.0032	2.207
ZEB1	14/803	0.0077	0.029	2.2
TBP	32/1843	1.54e-04	0.0032	2.191
IRF3	32/1865	1.87e-04	0.0033	2.165
ETS1	28/1661	5.38e-04	0.0052	2.126
E2F4	31/1847	3.24e-04	0.0040	2.117
NFYB	31/1852	3.38e-04	0.0041	2.111
MAFK	27/1616	7.40e-04	0.0065	2.107
ELF1	31/1875	4.10e-04	0.0046	2.085
TFAP2C	30/1822	5.24e-04	0.0052	2.076
PAX5	34/2072	2.73e-04	0.0039	2.069
CTCFL	35/2154	2.72e-04	0.0039	2.048
NFYA	30/1873	7.91e-04	0.0067	2.018
RFX5	29/1851	0.0013	0.0084	1.974
SP1	25/1611	0.0028	0.014	1.955
SP2	32/2063	9.27e-04	0.0072	1.954
TFAP2A	28/1810	0.0019	0.011	1.948
RELA	28/1815	0.0019	0.011	1.943
REST	28/1816	0.0019	0.011	1.942
E2F6	37/2432	6.12e-04	0.0056	1.916
MYOD1	42/2784	3.64e-04	0.0042	1.899
NFIC	16/1061	0.0164	0.046	1.899
GABPA	28/1864	0.00263	0.014	1.891
BACH1	27/1814	0.0034	0.017	1.874
FOS	54/3683	1.58e-04	0.0032	1.845
ZBTB7A	25/1735	0.0066	0.026	1.813

PBX3	28/1958	0.0049	0.021	1.799
HNF4A	34/2396	0.0026	0.014	1.785
STAT1	26/1855	0.0080	0.029	1.763
ZKSCAN1	26/1857	0.0081	0.029	1.761
ATF1	25/1833	0.0121	0.038	1.715
ELK1	25/1837	0.012	0.038	1.711
TCF7L2	35/2574	0.0044	0.020	1.709
YY1	36/2657	0.0042	0.020	1.703
STAT3	42/3450	0.012	0.037	1.528
IRF1	50/4204	0.011	0.036	1.493

FDR adjusted refers to Benjamini-Hochberg (B-H) False Discovery Rate correction for multiple testing.

Supplementary Table 6. Metabolic pathway enrichment analysis of AD-metabolic transcriptomics, proteomics and GWAS-orthologs datasets

Pathway	Transcr/ protein/ gene cover	Transcripts/proteins/genes mapped	P value (raw)	BH-adj P value
**Transcriptomics**				
Super-pathway of cholesterol biosynthesis	9/25	*Acat2, Dhcr24, Fdft1, Fdps, Hmgcs1, Hsd17b7, Nsdhl, Pmvk, Sqle*	8.72e-6	1.21e-3
Fatty acid α-oxidation	5/10	*Aldh1a7, Aldh3a1, Aldh3b1 Aldh7a1, Aldh9a1,*	1.60e-4	8.58e-3
Sucrose degradation	4/6	*Gpi, Hk1, Hk2, Hk3*	1.85e-4	8.58e-3
Fatty acid β-oxidation I	8/31	*Acaa2, Acadsb, Acadvl, Acsl5, Hadh, Hadhb, Peci, Slc27a2*	3.97e-4	1.05e-2
Putrescine degradation III	4/7	*Aldh1a7, Aldh7a1, Aldh9a1, Maob*	4.12e-4	1.05e-2
Cholesterol biosynthesis I	5/12	*Dhcr24, Fdft1, Hsd17b7, Nsdhl, Sqle*	4.54e-4	1.05e-2
Fatty acid β-oxidation IV	4/8	*Decr1, Decr2, Hadhb, Peci*	7.84e-4	1.56e-2
Tryptophan degradation III	5/15	*Acat2, Haao, Hadh, Ogdh, Tdo2*	1.48e-3	2.57e-2
Biosynthesis of prostaglandins	4/11	*Hpgd, Ptgds2, Ptgs1, Tbxas*	3.19e-3	4.44e-2
Cholesterol biosynthesis III	4/11	*Fdft1, Hsd17b7, Nsdhl, Sqle*	3.19e-3	4.44e-2
Epoxysqualene biosynthesis	2/2	*Fdft1, Sqle*	3.73e-3	3.74e-2
Fatty acid biosynthesis initiation III	2/2	*Fasn, Oxsm*	3.73e-3	3.74e-2
Fatty acid biosynthesis initiation II	2/2	*Fasn, Oxsm*	3.73e-3	3.74e-2
Phospholipases	7/34	*Pla2g4a, Pla2g4e, Pla2g5, Plce1, Plcg2, Plch1, Pld4*	3.73e-3	3.74e-2
**Proteomics**				
Aerobic respiration – electron donor II	21/72	*Ndufa11, Ndufa12, Ndufa3, Ndufa4, Ndufa5, Ndufa7, Ndufa9, Ndufab1, Ndufb11, Ndufb6, Ndufb7, Ndufs5, Ndufs6, Ndufs7, Ndufs8, Ndufv2, Sdha, Sdhb, Sdhc, Sdhd, Uqcrfs1*	9.26e-12	1.31e-9
NADH to cytochrome bo oxidase electron transfer	16/51	*Ndufa11, Ndufa 12, Ndufa3, Ndufa4, Ndufa5, Ndufa7, Ndufa9, Ndufab1, Ndufb11, Ndufb6, Ndufb7, Ndufs5, Ndufs6, Ndufs7, Ndufs8, Ndufv2*	9.78e-10	4.59e-8
NADH to cytochrome bd oxidase electron transfer	16/51	*Same enzymes* as NADH to cytochrome bo oxidase electron transfer	9.78e-10	4.59e-8
TCA cycle	8/13	*Aco1, Idh2, Sdha, Sdhb, Sdhc, Sdhd, Suclg1, Suclg2*	3.14e-8	1.11e-6
TCA cycle variation III	8/14	*Aco1, Idh3a, Sdha, Sdhb, Sdhc, Sdhd, Suclg1, Suclg2*	7.02e-8	1.98e-6
Aerobic respiration - electron donors reaction list	5/7	*Gpd1, Sdha, Sdhb, Sdhc, Sdhd*	5.39e-6	1.27e-4
Heme degradation	3/4	*Blvra, Blvrb, Hmox2*	4.57e-4	9.21e-3
Gluconeogenesis I	5/21	*Gapdh, Me1, Me3, Pgam1, Pgam2*	2.96 e-3	2.61e-2
Phosphatidyl-glycerol biosynthesis I	4/13	*Agpat1, Agpat4, Csd2, Lpcat4*	2.90 e-3	2.61e-2
CDP-diglycerol biosynthesis I	4/13	*Same enzymes* as phosphatidyl-glycerol biosynthesis I	2.90e-3	2.61e-2
Aspartate biosynthesis	2/2	*Got2, Pcx*	2.43e-3	2.63e-2
Cysteine biosynthesis/ Homocysteine degradation	2/2	*Cbs, Cth*	2.43e-3	2.63e-2
Cysteine biosynthesis II	2/2	*Same enzymes as* Cysteine biosynthesis/ Homocysteine degradation	2.43e-3	2.63e-2
Super-pathway of acetyl-CoA biosynthesis	3/6	*Acly, Dlat, Pdha1*	2.12e-3	2.99e-2
Glycolysis V	4/12	*Adpgk, Pgam1, Pgam2, Tpi1*	2.09e-3	2.99e-2
CDP-diglycerol biosynthesis II	4/12	*Same enzymes* as phosphatidyl-glycerol biosynthesis I	2.09e-3	2.99e-2

Phosphatidyl-glycerol biosynthesis II	4/4	*Same enzymes* as phosphatidyl-glycerol biosynthesis I	3.90e-3	3.24e-2
**GWAS-orthologs**				
Thyroid hormone metabolism I	2/3	*Dio1, Dio3*	8.91e-4	3.44e-2
Aerobic respiration – electron donor II	6/72	*Ndufa12, Ndufa2, Sdhb, Ndufc1, Ndufs3, Ndufs7*	1.40e-3	3.44e-2
NADH to cytochrome bo oxidase electron transfer	5/51	*Ndufa12, Ndufa2, Ndufc1, Ndufs3, Ndufs7*	1.74e-3	3.44e-2
NADH to cytochrome bd oxidase electron transfer	5/51	*Same enzymes* as NADH to cytochrome bo oxidase electron transfer	1.74e-3	3.44e-2

B-H adj. refers to Benjamini-Hochberg (B-H) FDR-adjusted p-value. Underlined genes/transcripts/proteins refer to omics elements that were mapped onto more than one metabolic pathway.

Supplementary Table 7. Unconditional and conditional EWCE analysis of AD-metabolic multi-omics datasets

Cell type	Fold change	SD from the mean	P value	B-H adj. p value	Condition
**Transcriptomics**	**dataset**				
Astrocytes (ependymal)	1.5460	7.266	0.0001	1.0e-07	Unconditional enrichment
Endothelial mural	0.7551	-3.408	1.000	1.000	Unconditional enrichment
Interneurons	0.7096	-4.418	1.000	1.000	Unconditional enrichment
Microglia	0.1491	5.769	0.0001	1.0e-07	Unconditional enrichment
Oligodendrocytes	1.0878	1.170	0.1268	0.2657	Unconditional enrichment
Pyramidal CA1 neurons	0.7164	-5.011	1.000	1.000	Unconditional enrichment
Pyramidal SS neurons	0.6804	-5.831	1.000	1.000	Unconditional enrichment
**Proteomics**	**dataset**				
Astrocytes (ependymal)	1.1626	2.0493	0.0272	0.0952	Unconditional enrichment
Endothelial mural	0.6887	-4.103	1.000	1.000	Unconditional enrichment
Interneurons	0.9915	-0.1187	0.5307	0.7921	Unconditional enrichment
Microglia	1.0772	0.8521	0.1941	0.4530	Unconditional enrichment
Oligodendrocytes	1.1988	2.4744	0.0100	0.0700	Unconditional enrichment
Pyramidal CA1 neurons	0.9701	-0.4914	0.6789	0.7921	Unconditional enrichment
Pyramidal SS neurons	0.9732	-0.4548	0.6703	0.7921	Unconditional enrichment
**GWAS-orthologs**	**dataset**				
Astrocytes (ependymal)	1.2336	1.7514	0.0480	0.3360	Unconditional enrichment
Endothelial mural	0.8842	-0.8969	0.8124	0.9478	Unconditional enrichment
Interneurons	0.9580	-0.3519	0.6150	0.8610	Unconditional enrichment
Microglia	1.0944	0.6165	0.2583	0.8165	Unconditional enrichment
Oligodendrocytes	1.0343	0.2531	0.3668	0.8165	Unconditional enrichment
Pyramidal CA1 neurons	1.0041	0.0397	0.4666	0.8165	Unconditional enrichment
Pyramidal SS neurons	0.7883	-2.1317	0.9908	0.9908	Unconditional enrichment
**Combined AD multi-omics**	**dataset**				
Astrocytes (ependymal)	1.3568	6.5462	0.0001	1.0e-07	Unconditional enrichment
Endothelial mural	0.7616	-4.567	1.000	1.000	Unconditional enrichment
Interneurons	0.8652	-2.821	0.9986	1.000	Unconditional enrichment
Microglia	1.1979	3.1434	0.0012	0.0056	Unconditional enrichment
Oligodendrocytes	1.1137	2.0620	0.0237	0.0737	Unconditional enrichment
Pyramidal CA1 neurons	0.8876	-2.7150	0.9976	1.000	Unconditional enrichment
Pyramidal SS neurons	0.8344	-4.1285	1.000	1.000	Unconditional enrichment
Astrocytes (ependymal)	1.3797	7.5402	0.0001	1.0e-07	Conditional enrichment (microglia controlled)
Endothelial mural	0.7668	-4.7705	1.000	1.000	Conditional enrichment (microglia controlled)
Interneurons	0.9222	-1.8281	0.9695	1.000	Conditional enrichment (microglia controlled)
Microglia	0.9998	-0.3555	0.6367	1.000	Conditional enrichment (microglia controlled)
Oligodendrocytes	1.1315	2.5311	0.0099	0.03885	Conditional enrichment (microglia controlled)
Pyramidal CA1 neurons	0.9359	-1.6967	0.9562	1.000	Conditional enrichment (microglia controlled)
Pyramidal SS neurons	0.8802	-3.3302	0.9999	1.000	Conditional enrichment (microglia controlled)
Astrocytes (ependymal)	0.9995	-1.5705	0.9424	1.000	Conditional enrichment (astrocyte controlled)
Endothelial mural	0.8188	-3.7026	0.9999	1.000	Conditional enrichment (astrocyte controlled)
Interneurons	0.9439	-1.2257	0.8891	1.000	Conditional enrichment (astrocyte controlled)
Microglia	1.2559	4.4759	0.0001	1.0e-07	Conditional enrichment (astrocyte controlled)
Oligodendrocytes	1.1245	2.3867	0.0111	0.03885	Conditional enrichment (astrocyte controlled)
Pyramidal CA1 neurons	0.9625	-0.9519	0.8268	1.000	Conditional enrichment (astrocyte controlled)
Pyramidal SS neurons	0.9185	-2.1752	0.9863	1.000	Conditional enrichment (astrocyte controlled)
Astrocytes (ependymal)	1.3459	6.7564	0.0001	1.0e-07	Conditional enrichment (oligodend. controlled)
Endothelial mural	0.7802	-4.4094	1.000	1.000	Conditional enrichment (oligodend. controlled)
Interneurons	0.9034	-2.2123	0.9884	1.000	Conditional enrichment (oligodend. controlled)
Microglia	1.1907	3.2431	0.0008	0.00448	Conditional enrichment (oligodend. controlled)

Oligodendrocytes	1.0000	0.0919	0.4409	1.000	Conditional enrichment (oligodend. controlled)
Pyramidal CA1 neurons	0.8928	-2.6396	0.9969	1.000	Conditional enrichment (oligodend. controlled)
Pyramidal SS neurons	0.8594	-3.5656	1.000	1.000	Conditional enrichment (oligodend. controlled)

SD refers to Standard Deviation, B-H adj. refers to Benjamini-Hochberg (B-H) FDR-adjusted p-value.

Table S8: GSMN and LIPID MAPS metabolite ID of lipid species predicted and validated in the AD predicted lipid signature. Please see Excel spread sheet Supplementary Table 8.

Table S9. Supplementary Table 9. Main lipid changes in ABCA7 mice identified by multivariate statistics. Please see Excel spread sheet Supplementary Table 9.

Table S10. Supplementary Table 10. Metabolites associated with Alzheimer's disease SNPs detected by ^{1}H NMR spectroscopy. Please see Excel spread sheet Supplementary Table 10.
